# Improvements in mental health associated with increased electronic communication and deterioration in physical health in adults aged 50+ during the COVID-19 pandemic

**DOI:** 10.3389/fpubh.2024.1369707

**Published:** 2024-06-21

**Authors:** Shay Musbat, Inbal Reuveni, Racheli Magnezi

**Affiliations:** ^1^Department of Management, Health Systems Management Program, Bar-Ilan University, Ramat Gan, Israel; ^2^Department of Psychiatry, Hadassah Hebrew University Medical Center, Ein Kerem, Jerusalem, Israel

**Keywords:** COVID-19 pandemic, physical health, mental health, depression, social networks, loneliness, electronic communication

## Abstract

**Background:**

Previous studies have documented changes in physical health, mental health and social parameters during COVID-19. At the same time, there are no comprehensive analyses of these parameters designed as longitudinal studies on large-scale older populations before and during the pandemic.

**Objective:**

This longitudinal study aims to provide a quantitative analysis of the COVID-19 impact on the physical, mental, and social parameters in adults aged 50 and older before, in the early stages, and during the COVID-19 pandemic.

**Methods:**

The data for this study were collected from three waves of the Survey of Health, Ageing and Retirement in Europe (SHARE), a supranational longitudinal database: pre-COVID (October 2019-March 2020), early-COVID (June-September 2020), and during-COVID (June-August 2021). The sample included 31,526 individuals, compared across the three-time points through nonparametric group comparison tests.

**Results:**

Physical health was subjectively rated as poorer in the during-COVID wave compared to the pre-COVID wave. Additionally, the number of illnesses or health conditions reported in the during-COVID wave was significantly higher than in the pre-COVID wave, with the biggest increases registered for cardiovascular diseases. The results also show that employment and overall social contact decreased while loneliness increased over time. Unexpectedly, mental health issues, such as sadness or depression and trouble sleeping, decreased significantly in the COVID waves compared to the pre-COVID wave. The analysis of two additional pre-COVID waves (2015, 2017) revealed that poorer pre-COVID mental health reflected in high values of sadness or depression and trouble sleeping was not an isolated peak but represented a typical baseline. The positive influence on the individuals’ mental health during COVID-19 was found to be electronic communication, which showed higher values than face-to-face communication and lowered the odds of sadness or depression.

**Conclusion:**

Future policies should thus consider the positive impact of electronic contacts on mental health to promote overall health in adults aged 50 and older.

## 1 Introduction

The Coronavirus Disease 2019 (COVID-19) pandemic has caused unprecedented disruption to all aspects of human life throughout the world. As of 2 May 2023, the worldwide number of confirmed cases of COVID-19 was over 687 million, with 6,866,733 deaths (1). To respond to this health emergency and contain the spread of COVID-19, governments enforced social distancing, workplace closures, cancelation of mass gatherings, and travel bans (2–4). These unparalleled restrictions had a massive impact on the population’s physical and mental well-being as well as their social behavior.

Research into previous severe epidemics with SARS-CoV-1 (severe acute respiratory syndrome coronavirus 1) and H1N1 (swine flu, is a subtype of influenza A virus) showed their negative impact on individuals’ physical and mental health (5–8). Similar COVID-19-related studies provide evidence of increased health consequences, particularly for older adults. These include higher rates of adverse morbidity with increased rates of hospitalization and death (up to 10% and above in older people) (9–11). Higher health risks such as COVID-19 for older people are associated with age-related comorbidities.

Among COVID-19 patients, more severe outcomes were likely to develop in older people and individuals with underlying medical conditions, such as cardiovascular disease, diabetes, chronic respiratory disease, or cancer (12). A COVID-19-related increase in the incidence of cardiovascular diseases can also be caused by physical inactivity (13). Physical activity is particularly important for diabetic patients, especially those with comorbid conditions (14). Forced inactivity has been found to be a severe threat to body homeostasis, increases the risk of insulin resistance in diabetic patients, and can lead to more severe COVID-19 outcomes, including death (15, 16). Underlying immune deficiencies are another risk factor for COVID-19 patients, such as cancer patients (17). When experiencing COVID-19, these patients are estimated to have a 3.5 times higher probability of severe events, such as mechanical ventilation, ICU (intensive care units) admission, and death (compared to non-cancer COVID patients) (18).

Additionally, numerous studies have documented an increase in adverse mental health outcomes during the pandemic, including anxiety, depression, and insomnia (19–21). These outcomes have been attributed to the decline in population mobility due to lockdown measures (22, 23). Based on available evidence, depressive symptoms, anxiety, and insomnia were higher in the youngest age group (18–35 years old). This can be due to the fact that workplace closures and related financial problems were more acute for younger people, thus leading to mental health disorders in this age group (24). In contrast to the above, other studies suggest that during the lockdown, symptoms of depression and sadness in adults gradually decreased (25).

Imposed restrictions also led to societal disruptions. Limited face-to-face interaction and social isolation during the pandemic (26) contributed to an increased feeling of loneliness (27), with loneliness viewed as an individual’s subjective perception of their social connections and relationships (28, 29). To compensate for the lack of in-person contact, electronic communication was increased (30). While being helpful in mitigating feelings of loneliness or depression, electronic communication has been found to produce mixed results (31, 32).

The pandemic also had an adverse effect on employment, with the International Labor Organization (33) reporting a 17.3% reduction in total working hours or 495 million full-time equivalent jobs in the second quarter of 2020 compared to the fourth quarter of 2019. One example of job loss implications is its effect on residence holding. Although in many European countries, a policy for temporary suspension of mortgage repayments was introduced for workers affected by the pandemic, only a few nations introduced similar policies for renters who typically have lower incomes and assets (34). In addition, many of the affected households did not have sufficient liquid assets to cover their potential losses (34).

Although previous studies have investigated the physical, mental, and social outcomes of the COVID-19 pandemic separately, there is a gap in research that comprehensively examines these parameters as a longitudinal study on a large-scale older population before and during the pandemic. Therefore, this longitudinal study aims to provide a quantitative analysis of the COVID-19 impact on the physical, mental, and social parameters in adults aged 50 and older before, in the early stages, and during the COVID-19 pandemic.

## 2 Methods

### 2.1 Data source: SHARE survey

The data for this study were collected from three survey cycles (waves) of the Survey of Health, Ageing and Retirement in Europe (SHARE) (35): Wave 8 (36)—pre-COVID wave (PCW, October 2019–March 2020); Wave 8 Corona Survey 1 (37)—early-COVID wave (ECW, June–September 2020) and Wave 9 Corona Survey 2 (38)—during-COVID wave (DCW, June–August 2021). In addition, upon receiving unexpected results when analyzing mental health parameters, we collected data for two additional pre-COVID waves: Wave 7 (39, 40)—2 years pre-COVID wave (2yPCW, 2017) and Wave 6 (41)—4 years pre-COVID wave (4yPCW, 2015; section 1 in the Supplementary material: SHARE questions used for the paper).

SHARE is a supranational longitudinal database of approximately 140,000 individuals aged 50 and older (around 530,000 interviews) from 26 European Union countries (excluding Ireland), Israel, and Switzerland. Being the largest European social science panel study, it engages the same respondents who participate over their life course and get measured on the same criteria. For data protection reasons, SHARE data are released in the form of “scientific-use files” protected by factual anonymity, and never contain identifying information about the SHARE participants (42). The current research is based on the SHARE data available to the public, with the respondents protected by factual anonymity and not personally identified. Therefore, being the analysis of secondary data provided by SHARE, this study does not require ethical approval or participants’ consent.

Since 2002, SHARE has been collecting objective data in the physical health sector (performance measurements, accelerometer measurements, blood samples) and in the economic sector (income, pension assets, administrative data). The SHARE database also collects individual-level data on social integration (social and family networks) and mental health (life satisfaction and well-being).

SHARE data collection methods underwent certain changes caused by the need to adapt to social distancing and other COVID-related measures. In the pre-COVID wave (October 2019–March 2020), data collection before March 2020 was carried out face-to-face as was standard in SHARE fieldwork—through a Computer-Assisted Personal Interview (CAPI). By early March, most of the fieldwork had been completed (around 70 percent of longitudinal interviews and 50 percent of refreshment interviews).

As the virus continued to spread, all SHARE fieldwork had to be suspended due to the lockdown. In order to continue collecting data on the health and living situation in Europe, SHARE replaced regular face-to-face interviews with Computer-Assisted Telephone Interviews (CATI) utilizing the new SHARE Corona questionnaire. This tool was a shorter version of the regular SHARE questionnaire, with more focus on COVID-19 living situations. The SHARE Corona questionnaire remained in use during the early-COVID and during-COVID waves, with data collected through CATI. CATI methodology allowed researchers to collect data in the same interviewer-administered mode (rather than self-administered mode) (43).

### 2.2 Sample

The analytic sample of participants included 31,526 individuals. The mean age of participants at the baseline wave (PCW) was 69.7 (standard deviation = 8.95). Participants were stratified by gender into four age groups: 50–59, 60–69, 70–79, and 80+ (Table 1).

**Table 1:** Basic demographics of participants: age and gender.

Characteristic	*N* (% gender; % age group)		
Age group	Male	Female	Both genders
50–59	1,565 (36.0)	2,783 (64.0)	4,348 (13.8)
60–69	5,042 (42.5)	6,824 (57.5)	11,866 (37.6)
70–79	4,503 (43.2)	5,928 (56.8)	10,431 (33.1)
80+	1,898 (38.9)	2,983 (61.1)	4,881 (15.5)
Total	13,008 (41.3)	18,518 (58.7)	31,526 (100)

The terminology employed above was drawn from the SHARE database, which, in addressing gender, differentiates between males and females rather than men and women. We acknowledge that gender identity is socially constructed, not biologically determined, and extends beyond a binary framework (44). However, we consider it fair to adhere to the original terminology used in the SHARE database. Consequently, this paper will adopt the same categorization of gender as employed by the SHARE database.

Being a panel study, SHARE allows to measure individual dynamics by engaging the same respondents. To select the sample for this study, we first considered those who were interviewed in all the waves under consideration. At the next stage, we limited the sample to the respondents aged 50 and above, excluding those who did not disclose their age at the time of the interview. Qualifying respondents were measured for their physical health, mental health, and social network parameters before, in the early stage, and during COVID-19 outbreak. To maintain a long-term consistency, if the participant refused to answer a question or gave a non-response to a question in at least one of the waves, all his/her responses to this question (in the remaining waves) were excluded from the analysis. Table 2 shows the study parameters under analysis, distribution of answers in each applicable wave, and the statistical significance of the difference for each answer option between the waves.

**Table 2:** Study parameters, answer options and distribution of responses.

Study parameters	COVID wave			χ^2^ (McNemar test)
	Pre, *N* (%)	Early, *N* (%)	During, *N* (%)	
Physical Health Parameters				
*Rating of subjective health*				
Poor	2,874 (9.1)	N/A^1^	3,002 (9.5)	5.380*^2^
Fair	9,308 (29.6)	N/A	9,585 (30.5)	8.810**
Good	12,347 (39.3)	N/A	12,644 (40.2)	8.288**
Very good	5,264 (16.7)	N/A	4,855 (15.4)	29.699***
Excellent	1,668 (5.3)	N/A	1,375 (4.4)	46.114***
*Illness or health conditions*				
Heart attack or other heart problem	3,971 (12.7)^3^	N/A	5,506 (17.6)	567.436***
High blood pressure or hypertension	14,644 (46.9)	N/A	15,908 (51.0)	277.132***
Diabetes or high blood sugar	4,532 (14.5)	N/A	5,334 (17.1)	296.763***
Chronic lung disease	1,800 (5.8)	N/A	2,104 (6.7)	49.842***
Cancer or malignant tumor	1,536 (4.9)	N/A	1,742 (5.6)	26.070***
Hip fracture or femoral fracture	511 (1.6)	N/A	772 (2.5)	77.970***
Mental Health Parameters				
*Sad or depressed last month*				
Yes	12,108 (39.1)	7,663 (24.8)	9,130 (29.5)	P-E^4^: 1,994.258*** P-D: 894.302*** E-D: 275.074***
No	18,828 (60.9)	23,273 (75.2)	21,806 (70.5)	-
*Trouble sleeping recently*				
Yes	11,371 (36.6)	8,396 (27.0)	9,828 (31.7)	P-E: 1,020.030*** P-D: 275.811*** E-D: 266.081***
No	19,675 (63.4)	22,650 (73.0)	21,218 (68.3)	-
Social Parameters				
*Current employment situation*				
Working	5,773 (18.5)	4,970 (15.9)	4,864 (15.6)	P-E: 254.734*** P-D: 419.575*** E-D: 4.208
Not working	25,495 (81.5)	26,298 (84.1)	26,404 (84.4)	-
*Face-to-face and/or electronic contact* ^5^				
*Feels lonely*				
Hardly ever or never	22,559 (72.9)	22,165 (71.6)	21,267 (68.7)	P-E: 21.895*** P-D: 224.439*** E-D: 118.325***
Some of the time	6,371 (20.6)	6,655 (21.5)	7,342 (23.7)	P-E: 10.616** P-D: 118.218*** E-D: 63.465***
Often	2,013 (6.5)	2,123 (6.9)	2,334 (7.6)	P-E: 4.749* P-D: 38.980*** E-D: 17.963***

^1^Data for ECW were excluded from the analysis as the illnesses or health conditions question elicited almost 93% of “*Not applicable*” responses in this wave. ^2^Significance level: *p < 0.05, **p < 0.01, ***p < 0.001. For mental health and social parameters, the significance level was set at *p* < 0.017 to account for multiple comparisons. ^3^In the Illness or health conditions subsection, the percentage in each line is calculated as the proportion of the total sample (31,216). Please note that the same participant could select more than one health condition, or none of them. ^4^P, E and D represent Pre, Early, and During COVID waves (respectively). ^5^For simplicity, the table disregards original categories presented in response options to the questions on face-to-face and/or electronic communication. For details, please see the “Social parameters” section.

### 2.3 Measures

#### 2.3.1 Physical health parameters

Physical health parameters were measured in participants of PCW and DCW (subsection 1.1 in the Supplementary material: Physical health parameters-related questions). Participants’ subjective health status was measured using a single-item question: “*Would you say your health is…*.” Participants were offered five-point rating scale response options: “*Excellent*” [1], “*Very good*” [2], “*Good*” [3], “*Fair*” [4], or “*Poor*” [5]. Throughout the study, the scores were adjusted for analysis (“*Excellent*” scored as 5, “*Very good*” scored as 4, etc.). The scores for both waves were analyzed. The subjective health status question in ECW was excluded from analysis, as it referred to the past “*Before the outbreak of Corona, would you say your health is…*.”

The information on the illnesses or health conditions was collected by SHARE using multiple choice questions: “*Has a doctor ever told you that you had/Do you currently have any of the conditions on this card? With this we mean that a doctor has told you that you have this condition, and that you are either currently being treated for or bothered by this condition*” (for PCW), and “*Do you have any of the following illnesses or health conditions? With this we mean that a doctor has told you that you have this condition, and that you are either currently being treated for or bothered by this condition*” (for ECW and DCW). Data for ECW were excluded from the analysis as the illnesses or health conditions question elicited almost 93% of “*Not applicable*” responses. One reason for such responses could hypothetically be the respondents’ lack of access to standard healthcare services at the early-COVID stage. Numerous studies of that period report delays, postponements and cancelations in many regular medical visits—caused by both subjective (individual fear) and objective factors (such as significant shortages of medical staff) (45–47). As a result, survey respondents could have no medical attention and receive no treatment, thus having no diagnoses to report and opting for the “*Not applicable*” answer. The picture in PCW and DCW was different. For analysis, we selected six response options common for both waves, PCW and DCW: “*Heart attack or other heart problem*,” “*High blood pressure or hypertension*,” “*Diabetes or high blood sugar*,” “*Chronic lung disease*,” “*Cancer or malignant tumor*,” “*Hip fracture or femoral fracture.”* The number of occurrences of the six illnesses or health conditions above in each wave was summed up for each respondent and analyzed. In addition, each of the illnesses or health conditions above was analyzed by its prevalence (as a proportion) in PCW compared to DCW.

#### 2.3.2 Mental health parameters

Mental health parameters in participants across the three waves, PCW, ECW, and DCW [subsection 1.2 in the Supplementary material: Mental health parameters-related questions (2019–2021)], were measured with two binary choice questions (about feeling sad/depressed and about sleep quality): question 1: “*In the last month, have you felt sad or depressed*?” (with “*Yes*”/“*No*” answers), and question 2: “*Have you had trouble sleeping recently?*” The two response options to question 2 were “*Trouble sleeping or recent change in sleep pattern*” or “*No trouble sleeping*.” Questions 1 and 2 above were analyzed separately.

#### 2.3.3 Social parameters

The current employment situation was measured across the three waves (subsection 1.3 in the Supplementary material: Social parameters-related questions). For PCW and DCW, the survey used a multiple-choice question: “*Which of the following best describes your current employment situation?*” Participants were offered six response options: “*Retired*,” “*Employed or self-employed*,” “*Unemployed*,” “*Permanently sick or disabled*,” “*Homemaker*” or “*Other*.” For the current study, the “*Employed or self-employed*” option was interpreted as working, while other options (i.e., “*Retired*,” etc.) were interpreted as not working. For ECW, the survey used a binary choice question: “*Due to the Corona crisis have you become unemployed, were laid off or had to close your business?*” with the response options being “*Yes*” or “*No*.” For the current study, individuals who answered “*No*” were considered working, and individuals who answered “*Yes*” were considered not working. In addition, responses of the subjects who reported not working before the ECW (i.e., due to being “*Retired*,” “*Permanently sick or disabled*,” etc.) were categorized by the SHARE as “*Not applicable*.” For the purposes of our analysis, these subjects were considered not working. For each of the waves, PCW, ECW, and DCW, all the responses were analyzed as a dichotomous variable: “Working” or “Not working” over time.

The participants’ feeling of loneliness, as a subjective perception of social connections and relationships across the three waves, was measured with a single-item question: “*How much of the time do you feel lonely?*” Participants were offered three-point rating scale response options (with adjusted scores): “*Hardly ever or never*” [1], “*Some of the time*” [2], or “*Often*” [3]. The scores for the three waves, PCW, ECW, and DCW, were analyzed.

To measure the subjects’ social integration, the SHARE survey included two separate multiple-choice questions on face-to-face and/or electronic contacts for ECW and DCW. Question 1 referred to face-to-face contact: “…*how often did you have personal contact, that is, face-to-face, with the following people from outside your home?*” with two temporal modifications: “s*ince the outbreak of Corona…*” (for ECW) and “*during the last three months…*” (for DCW). Question 2 referred to electronic communication: “…*how often did you have contact by phone, email or any other electronic means with the following people from outside your home?*” with the same two temporal modifications: “*since the outbreak of Corona…*” (for ECW) and “*during the last three months…*” (for DCW). The five response options with adjusted scores (for both questions) were: “*Daily*” [5], “*Several times a week*” [4], “*About once a week*” [3], “*Less often*” [2], or “*Never*” [1] *—* applied to each of the categories: “*Own children*,” “*Own parents*,” “*Other relatives*,” “*Other non-relatives like neighbours, Friends, or colleagues*.”

PCW participants were asked a combined question about their face-to-face and electronic communication: “*During the past twelve months, how often did you have contact with*”; spouse, family members, children, siblings, parents, friends, formal helpers, or others in social network (average contact) “*either in person, by phone or mail, email or any other electronic means?*” The seven response options with adjusted scores were: “*Daily contact*” [7], “*Several times/week*” [6], “*1/week*” [5], “*Every 2 weeks*” [4], “*Once a month*” [3], “*Less than once a month*” [2], or “*Never*” [1].

To standardize the seven response options in PCW, with the five response options in ECW and DCW, and merge them into a common scale of five response options, the following grouping was applied: “*Daily*” [5], “*Several times a week*” [4], “*About once a week*” [3], “*Less often*”/“*Every 2 weeks*”/*“Once a month*”/“*Less than once a month*” [2], “*Never*” [1]. This grouping allowed for a consistent comparison and analysis of the data across the three waves.

The answers received from the participants in response to the social integration questions above were analyzed combined (face-to-face and electronic communication) for both questions (for PCW, ECW, and DCW) and separately (face-to-face or electronic communication) for each of the two questions (for ECW and DCW). For combined analysis, the subject’s contacts were categorized as “*Children*,” “*Parents*,” “*Other relatives*,” and “*Neighbors, friends or colleagues*.” For PCW, the “*Other relatives*” category included the subcategories “*Spouse*,” “*Family members*,” and “*Siblings*.” The highest score received by any of these subcategories was applied as the score of the entire category. The “*Neighbors, friends or colleagues*” category included the subcategories “*Friends*,” “*Formal helpers*,” and “*Others*.” The highest score received by any of these subcategories was applied as the score of the entire category. The analysis for ECW and DCW included four categories of contacts found in both waves: “*Children*,” “*Parents*,” “*Other relatives*” and “*Neighbors, friends or colleagues*.” In PCW, the social integration question did not differentiate between face-to-face and electronic communication, unlike ECW and DCW, where the questions were asked separately. For this reason, to be able to compare PCW scores in this question with those of the other two waves, ECW and DCW, the higher of the two scores received for social interaction (face-to-face or electronic) for ECW and DCW in each contact category for each respondent was applied as the score of the entire category. To maintain a long-term consistency, if no answer appeared in a contact category for at least one of the waves (PCW, ECW, or DCW; score 0), the respondent’s scores for this category were excluded from the analysis. The scores for all the contact categories, for each respondent, were summed up for each of the waves, PCW, ECW, and DCW, and analyzed.

Separate analysis was applied to participants’ responses to the social integration questions only for ECW and DCW (as the social integration question in PCW did not differentiate between face-to-face and electronic communication). For analysis, we selected the subject’s contacts that appeared in both waves, ECW and DCW — these were “*Children*,” “*Parents*,” “*Other relatives*,” and “*Neighbors, friends or colleagues*.” The analysis was carried out for face-to-face and electronic communication separately using the selected contact categories in each of the waves (ECW and DCW). To maintain a long-term consistency, if no answer appeared in a contact category (separately for face-to-face or electronic communication; score 0) for at least in one of the two waves, the respondent’s scores for this category were excluded from the analysis. The scores for all the contact categories for each respondent were summed up for each of the two waves, ECW and DCW, separately (for face-to-face and electronic communications) and analyzed.

At the next stage, we checked for possible associations between having electronic contacts and feeling sad or depressed in ECW and DCW. For this, we classified all responses to the question about the frequency of electronic contacts into two categories: those reporting electronic contacts (with varying frequency) and those reporting no electronic contacts. For each individual, we looked into the relationship between having / not having electronic communication and feeling / not feeling sad or depressed. “*Not applicable*” responses to the electronic contacts question—if they appeared in all contact categories for the given respondent—were not considered in the analysis.

### 2.4 Data analysis

The analysis was carried out using SPSS, version 25.0 (IBM Corp., Armonk, NY, United States).

To assess the significance of changes observed in physical health parameters between PCW and DCW, the study employed the Wilcoxon Signed-Rank test. This non-parametric statistical method is used to compare two sets of scores from the same participants in order to assess differences in population mean ranks from one time point to another (48). Further, to compare the proportions of each type of illness or health condition between PCW and DCW and determine the statistical significance of changes, we used the McNemar test. The McNemar test is a non-parametric statistical method used to measure the differences between two related groups on a dichotomous dependent variable (49). The *p*-value of <0.05 was used to determine statistical significance.

To determine if there are significant differences in mental health parameters between PCW, ECW, and DCW, the study employed Cochran’s Q test. This is a non-parametric statistical method which is used for nominal dichotomous data when there are more than two related groups (50). To determine the significance of difference between pairs of waves, the study employed the McNemar test with Bonferroni Correction as a post-hoc analysis. The significance level was set at *p* < 0.017 to account for multiple comparisons.

For social parameters, the study ran Cochran’s Q test to establish significant differences in the current employment situation among the three waves. The significance of difference between pairs of waves was analyzed with the McNemar test with Bonferroni Correction as a post-hoc analysis. For underrepresented categories with few reported responses, the analysis used binomial distribution. To examine significant changes in participants’ feelings of loneliness as a subjective perception of social connections and their social integration (face-to-face and electronic social communication through combined analysis, see Measures above) across the three waves, we conducted the Friedman test. The Friedman test is a non-parametric statistical method to assess the differences between three or more related groups (51). The Wilcoxon Signed-Rank Test with Bonferroni Correction as a post-hoc analysis was employed to determine the significance of difference between pairs of waves. The significance level was set at *p* < 0.017 to account for multiple comparisons. As for the separate analysis of the data on social integration (face-to-face or electronic communication) between ECW and DCW, it was carried out through the Wilcoxon Signed-Rank test. In addition, to test for a significant association between using electronic communication and feeling sad or depressed, we conducted Pearson’s Chi-square test [χ^2^(degrees of freedom)]. Pearson’s Chi-square test (or the chi-square test of association) is a non-parametric statistical method to determine if there is a relationship between two categorical variables (52). The strength of the association was measured through an odds ratio (OR) analysis with a 95% confidence interval (CI). Odds ratio is a measure of association between an exposure and an outcome (53). Pearson’s Chi-square test and OR analysis were conducted for ECW and DCW.

For instances where the post-hoc statistical analysis results by gender were overlapping, we reported a general range instead of providing a specific range for each gender.

It has to be repeated that SHARE data collection methods had to adapt to social distancing and other COVID-related measures. For that reason, for the early-COVID and during-COVID waves, a Computer-Assisted Personal Interview was replaced with a Computer-Assisted Telephone Interview (43). This switch from face-to-face to telephone interviews could potentially affect the results. Among the challenges presented by telephone interviews are shorter attention spans, which means they should be kept shorter compared to face-to-face interviews. To minimize this challenge, the SHARE team shortened the questionnaire administered in respective waves (43). Another potential effect of switching from face-to-face to telephone interviews could be lower data quality, as respondents may be less honest in face-to-face than telephone interviews (54). However, SHARE is a longitudinal study, with repeated observations of the same individual on the same variables. This approach minimizes the probability of reducing data quality. Further, a comparison of face-to-face interviewing with telephone interviewing in a qualitative study revealed no significant differences in the interviews—after comparing the interview transcripts (54). This suggests that with a change in the SHARE data collection methods, the results of the analysis we undertook still hold. Additional considerations strengthening our viewpoint are twofold. First, both face-to-face and telephone interviews represent the same mode of data collection: interviewer-administered as opposed to self-administered. Second, both of these types of interviews are computer assisted and use SHARE software tools. Based on the review from the SHARE Central Coordination Team, these tools had been installed on the interviewers’ laptops before the lockdowns (at the start of wave 8), so the interviewers were able to continue using them by telephone during the lockdowns (43). Based on this, the data collected across the three waves under study can still be considered comparable, and its quality did not suffer as a result of changes in SHARE data collection methods.

## 3 Results

### 3.1 Physical health parameters

The subjective health rating score for individuals in DCW (mean rank 7,350.93) was significantly lower than in PCW (mean rank 7,475.27, *p* < 0.001). This applies to both genders aged 50–59, and 70 and above, and to females aged 60–69 (from *p* = 0.047 to *p* < 0.001; subsection 2.1.1 in the Supplementary material: Changes in rating of subjective health). The average subjective health rating score reported in DCW was lower compared to that of PCW by 1.7%. Similarly, the number of illnesses or health conditions for individuals in DCW (mean rank = 5,770.91) was significantly higher than in PCW (mean rank = 5,495.61, *p* < 0.001). This applies to all age groups for both genders (*p* < 0.001; subsection 2.1.2 in the Supplementary material: Changes in the number of illnesses or health conditions). The average number of illnesses or health conditions reported in DCW was higher compared to that of PCW by 16.2%.

**Figure 1:**
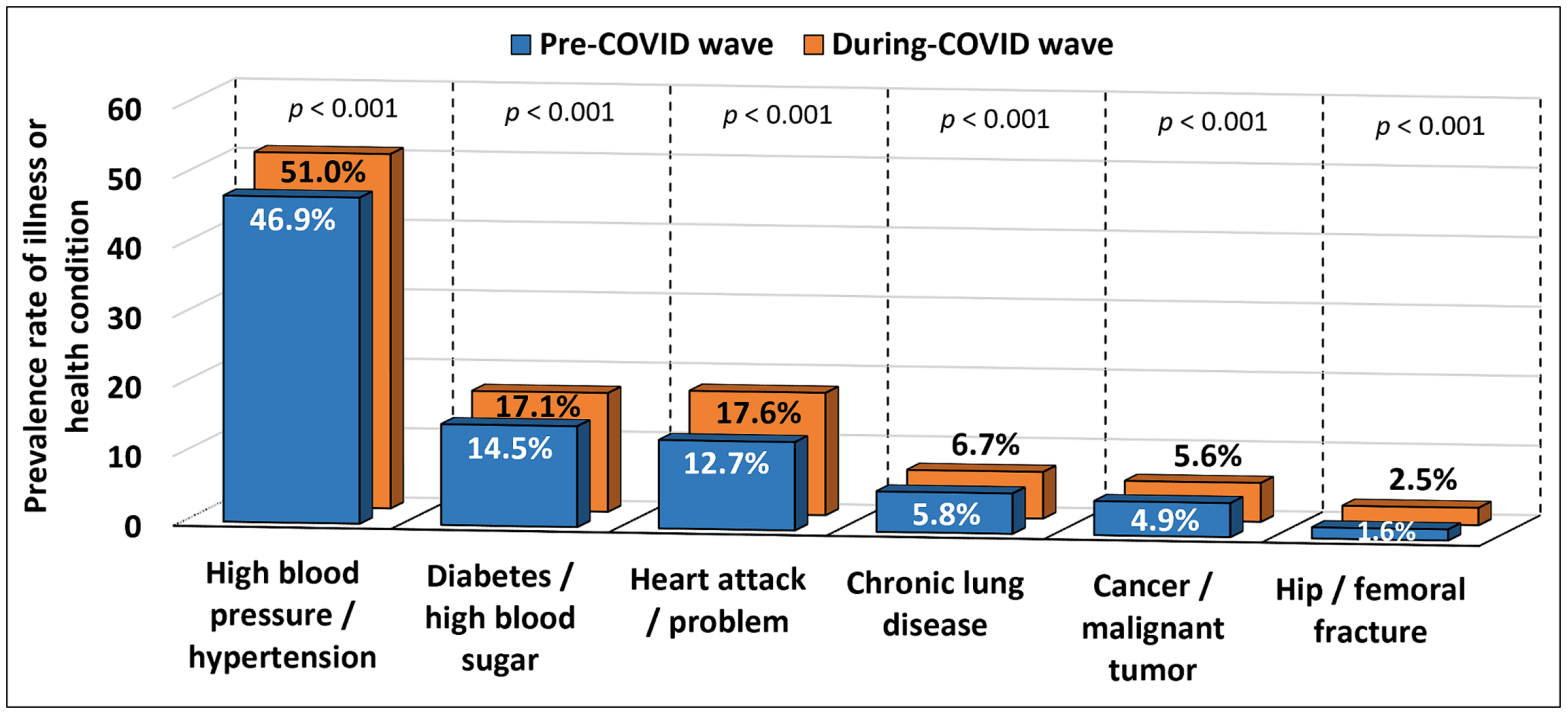
Prevalence rates of illness or health condition in the total population in PCW and DCW.

Examination of the percentages of prevalence rates between PCW and DCW revealed significant differences for all illnesses and health conditions (all *p* < 0.001; Figure 1).

The biggest increase in the prevalence rate was observed for heart attack or other heart problems (4.9%). The increase was registered for all age groups for both genders (*p* < 0.001). A slightly lower increase was observed for high blood pressure or hypertension (4.1%), with the increase registered for both males and females aged 50–79 and males aged 80+ (from *p* = 0.019 to *p* < 0.001). The analysis also showed an increased prevalence rate for diabetes or high blood sugar (2.6%) across all age groups for both genders (*p* < 0.001). As for an increase in the prevalence rate for chronic lung disease (0.9%), it was registered for both males and females aged 50–69 and males aged 70–80+ (from *p* = 0.035 to *p* < 0.001). A similar increase was also observed in the prevalence rate for hip fracture or femoral fracture (0.9%) among both males and females aged 60–80+ (from *p* = 0.042 to *p* < 0.001). The lowest increase in the prevalence rate was registered for cancer or malignant tumor (0.7%) among males aged 60–79 and females aged 70–79 (from *p* = 0.045 to *p* = 0.003; subsection 2.1.3 in the Supplementary material: Changes in the type of illness or health condition).

### 3.2 Mental health parameters

The analysis revealed a significant difference in the proportion of individuals who reported feeling sad or depressed across the three waves (*p* < 0.001). The number of sad or depressed individuals decreased from 12,108 (39.1%) in PCW to 7,663 (24.8%) in ECW and increased to 9,130 (29.5%) in DCW. There was a significant difference in the number of individuals feeling sad or depressed between PCW and ECW (*p* < 0.001), between PCW and DCW (*p* < 0.001), and between ECW and DCW (*p* < 0.001).

A significant difference was found among all age groups for both genders (*p* < 0.001), where the number of sad or depressed individuals decreased from PCW to ECW and then increased in DCW (but being still below the PCW values). There was a significant difference in the number of subjects feeling sad or depressed among all age groups for both genders between PCW and ECW (*p* < 0.001), between PCW and DCW (*p* < 0.001), and between ECW and DCW [from *p* = 0.005 to *p* < 0.001; subsection 2.2.1 in the Supplementary material: Changes in sadness or depression across three waves (PCW, ECW and DCW)].

To determine whether the number of sad or depressed individuals peaked in PCW and then decreased to baseline in ECW and DCW or was itself the baseline and then decreased in ECW and DCW, it was decided to analyze an earlier period (2017) as an additional wave before PCW (2yPCW). The data for 2yPCW was only collected for shared individuals from PCW, ECW, and DCW (*N* = 5,715). It was found that the number of sad or depressed individuals in 2yPCW was as high as in PCW and not significantly different, and both were higher and significantly different from ECW and DCW [subsection 2.2.2 in the Supplementary material: Changes in sadness or depression across four waves (2yPCW, PCW, ECW and DCW)]. However, since the question about feeling sad or depressed elicited few responses among the 2yPCW subjects (only 18% of them answered “*Yes*” or “*No*”), it was decided to analyze a wave immediately before it — 4yPCW [2015; subsection 1.4 in the Supplementary material: Mental health parameters-related questions (2015–2017)]. In 4yPCW, 95% of the total pool of participants provided “*Yes*” or “*No*” answers to the sadness or depression question. The data for 4yPCW was only collected for shared individuals from PCW, ECW, and DCW (*N* = 21,283).

**Figure 2:**
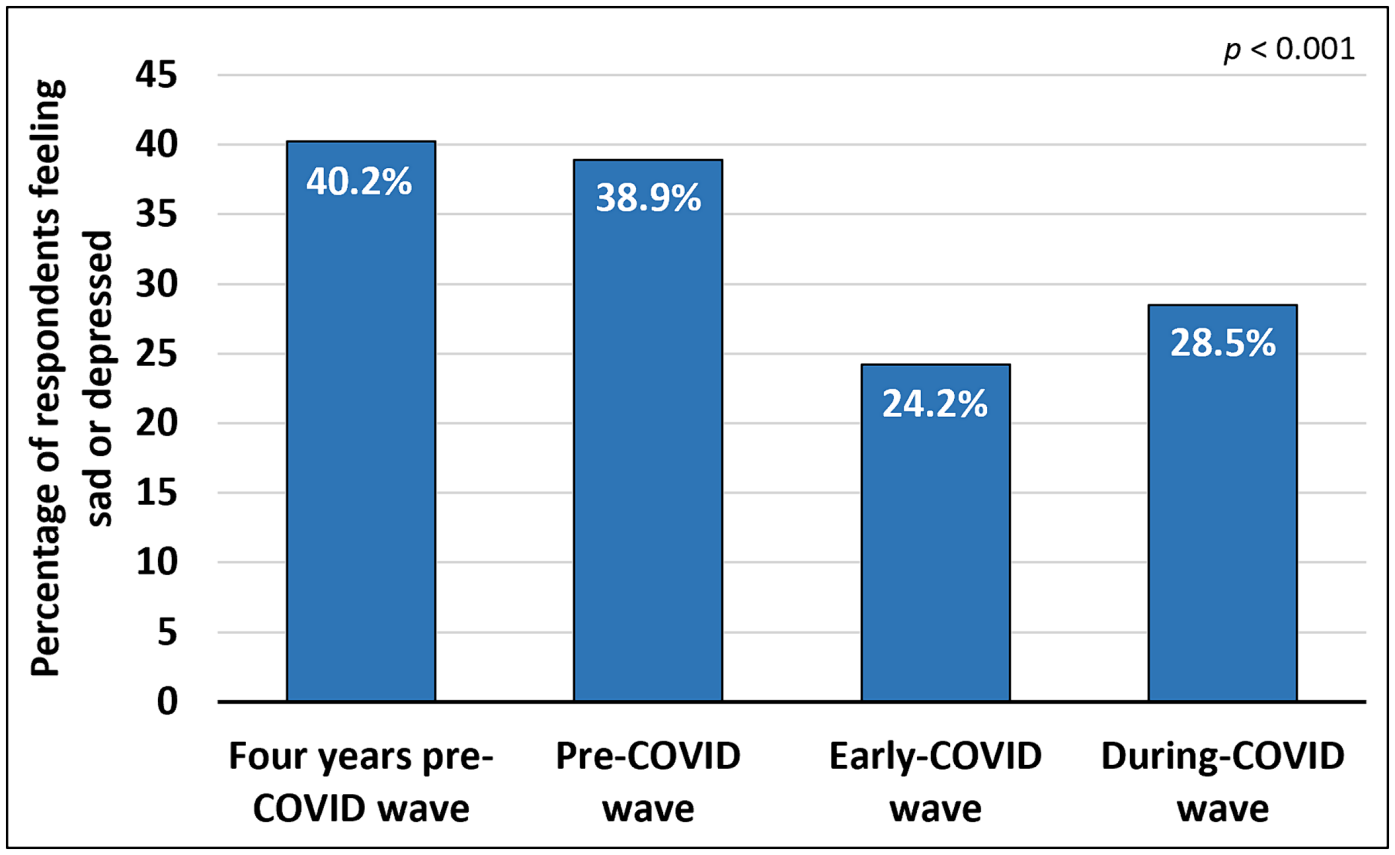
Percentage of the total population feeling sad or depressed in 4yPCW, PCW, ECW and DCW.

The analysis revealed a significant difference in the proportion of individuals who reported feeling sad or depressed across the four waves—4yPCW, PCW, ECW, and DCW (*p* < 0.001). The number of sad or depressed individuals decreased from 8,563 (40.2%) in 4yPCW to 8,280 (38.9%) in PCW to 5,155 (24.2%) in ECW and increased to 6,067 (28.5%) in DCW (Figure 2).

There was a significant difference in the number of subjects feeling sad or depressed between 4yPCW and PCW (*p* = 0.001), between 4yPCW and ECW (*p* < 0.001), between 4yPCW and DCW (*p* < 0.001), between PCW and ECW (*p* < 0.001), between PCW and DCW (*p* < 0.001), and between ECW and DCW (*p* < 0.001). The significance level was set at *p* < 0.008 to account for multiple comparisons [subsection 2.2.3 in the Supplementary material: Changes in sadness or depression across four waves (4yPCW, PCW, ECW and DCW)].

The analysis revealed a significant difference in the proportion of individuals who reported trouble sleeping or recent changes in the sleep pattern (*p* < 0.001). The number of individuals who reported trouble sleeping decreased from 11,371 (36.6%) in PCW to 8,396 (27.0%) in ECW and increased to 9,828 (31.7%) in DCW. There was a significant difference in the number of individuals who reported trouble sleeping between PCW and ECW (*p* < 0.001), between PCW and DCW (*p* < 0.001), and between ECW and DCW (*p* < 0.001).

A significant difference was found among all age groups for both genders (*p* < 0.001), where the number of individuals who reported trouble sleeping decreased from PCW to ECW and then increased in DCW (but being still below the PCW values). There was a significant difference in the number of subjects who reported trouble sleeping between PCW and ECW among all age groups for both genders (*p* < 0.001), between PCW and DCW among ages 60–79 for both genders, 50–59 and 80+ for females (*p* < 0.001), and between ECW and DCW among all age groups for both genders [*p* < 0.001; subsection 2.2.4 in the Supplementary material: Changes in trouble sleeping or recent change in sleep pattern across three waves (PCW, ECW and DCW)].

To determine whether the number of individuals who reported trouble sleeping peaked in PCW and then decreased to the baseline in ECW and DCW or was itself the baseline and then decreased in ECW and DCW, 2yPCW was analyzed. The data for 2yPCW was only collected for shared individuals from PCW, ECW, and DCW (*N* = 5,746). It was found that the number of individuals who reported trouble sleeping in 2yPCW was as high as in PCW and not significantly different, and both were higher and significantly different from ECW and DCW [subsection 2.2.5 in the Supplementary material: Changes in trouble sleeping or recent change in sleep pattern across four waves (2yPCW, PCW, ECW and DCW)]. However, since the question about trouble sleeping or recent change in sleep pattern elicited few responses among the 2yPCW subjects (only 18% of them answered “*Yes*” or “*No*”), it was decided to analyze 4yPCW. In 4yPCW, 96% of the total pool of participants provided “*Yes*” or “*No*” answers to the sleep patterns question. The data for 4yPCW was only collected for shared individuals from PCW, ECW and DCW (*N* = 21,356).

**Figure 3:**
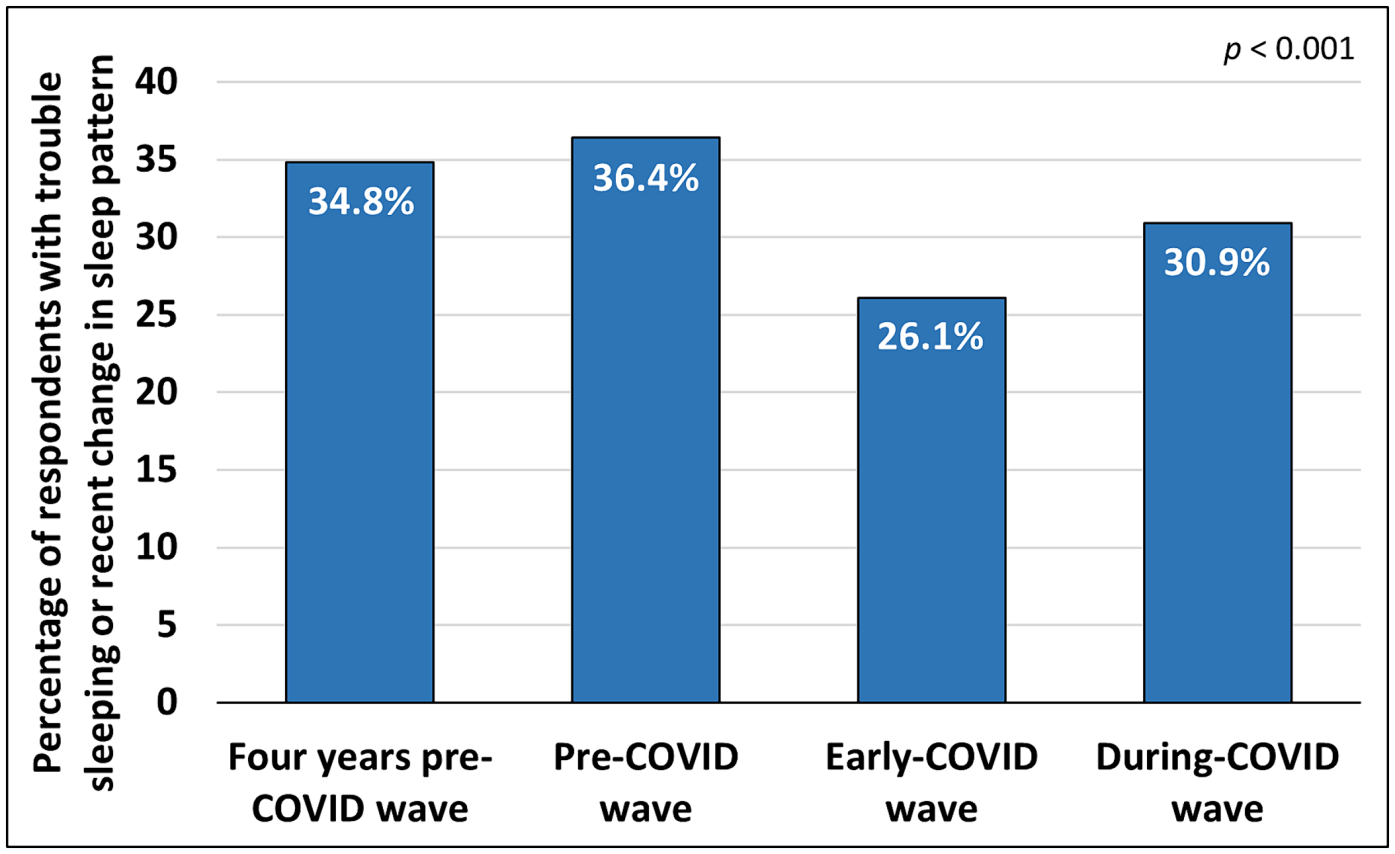
Percentage of the total population with reported trouble sleeping in 4yPCW, PCW, ECW and DCW.

The analysis revealed a significant difference in the proportion of individuals who reported trouble sleeping or recent changes in the sleep pattern across the four waves - 4yPCW, PCW, ECW, and DCW (*p* < 0.001). The number of individuals who reported trouble sleeping increased from 7,439 (34.8%) in 4yPCW to 7,777 (36.4%) in PCW, decreased to 5,568 (26.1%) in ECW, and increased to 6,601 (30.9%) in DCW (Figure 3).

There was a significant difference in the number of subjects who reported trouble sleeping between 4yPCW and PCW (*p* < 0.001), between 4yPCW and ECW (*p* < 0.001), between 4yPCW and DCW (*p* < 0.001), between PCW and ECW (*p* < 0.001), between PCW and DCW (*p* < 0.001) and between ECW and DCW (*p* < 0.001). The significance level was set at *p* < 0.008 to account for multiple comparisons [subsection 2.2.6 in the Supplementary material: Changes in trouble sleeping or recent change in sleep pattern across four waves (4yPCW, PCW, ECW and DCW)].

### 3.3 Social parameters

The analysis of the subjects’ current employment situation revealed a significant difference in the proportion of individuals who reported working across the three waves (*p* < 0.001). The number of working individuals decreased from 5,773 (18.5%) in PCW to 4,970 (15.9%) in ECW and 4,864 (15.6%) in DCW. There was a significant difference in the number of individuals who reported working between PCW and ECW (*p* < 0.001) and between PCW and DCW (*p* < 0.001). However, the number of working individuals in ECW was not significantly different from those who reported working in DCW (*p* = 0.040).

**Figure 4:**
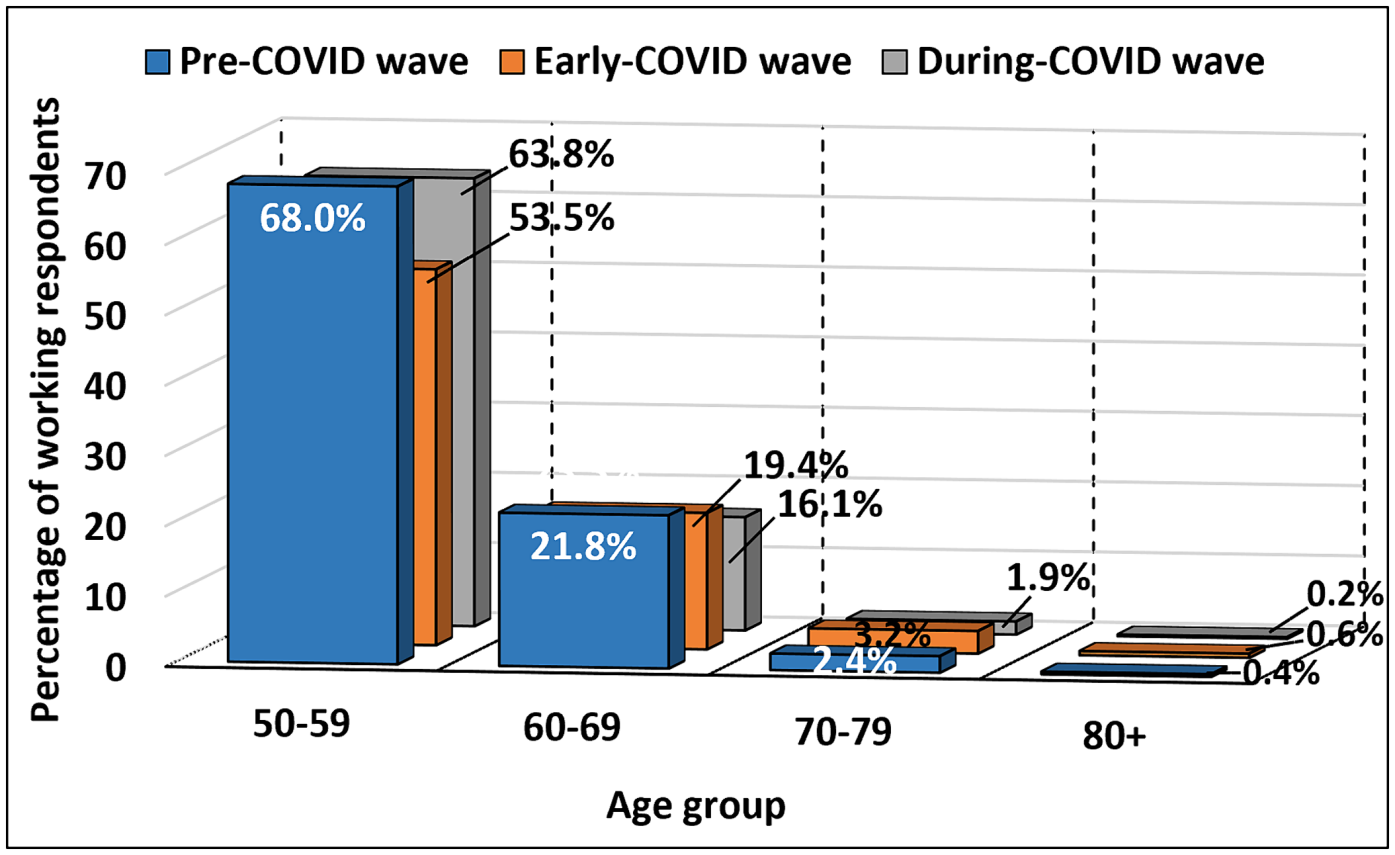
Percentage of working respondents by age group in PCW, ECW and DCW.

A significant difference was found among ages 50–79 for both genders and 80+ for males (from *p* = 0.002 to *p* < 0.001). The number of individuals of both genders aged 50–79 and in males aged 80+ who reported working decreased from PCW to ECW and then increased in DCW (but being still below the PCW values). In the 60–69 age group, including both genders, the number of those who reported working decreased steadily throughout the waves. There was a significant difference in the number of individuals who reported working between PCW and ECW among ages 50–69 for both genders and 70–79 for males (*p* < 0.001), between PCW and DCW among ages 50–69 for both genders and 70–79 for females (from *p* = 0.001 to *p* < 0.001), and between ECW and DCW among ages 50–79 for both genders and 80+ for males [from *p* = 0.003 to *p* < 0.001; subsection 2.3.1 in the Supplementary material: Changes in current employment situation across three waves (PCW, ECW and DCW)]. Changes in the percentage of working respondents in each age group across the three waves (pre-COVID, early-COVID and during-COVID) are shown in Figure 4 and detailed in Table 3.

**Table 3:** Number and percentage of working respondents by age group in PCW, ECW and DCW.

Age group	*N* (% age group)			
	Pre-COVID wave	Early-COVID wave	During-COVID wave	Total age group
50–59	2,937 (68.0%)	2,312 (53.5%)	2,756 (63.8%)	4,322 (100%)
60–69	2,574 (21.8%)	2,296 (19.4%)	1,904 (16.1%)	11,818 (100%)
70–79	244 (2.4%)	334 (3.2%)	193 (1.9%)	10,376 (100%)
80+	18 (0.4%)	28 (0.6%)	11 (0.2%)	4,752 (100%)

The analysis revealed a significant difference in feeling lonely scores in the individuals across the three waves (*p* < 0.001). The mean rank scores of feeling lonely increased from 1.97 in PCW to 1.99 in ECW and 2.04 in DCW. There was a significant difference in the feeling lonely scores between PCW and ECW (*p* < 0.001), between PCW and DCW (*p* < 0.001), and between ECW and DCW (*p* < 0.001). The average feeling lonely score reported in DCW was higher by 2.7% compared to ECW, which in its turn was higher by 1.2% compared to PCW.

**Figure 5:**
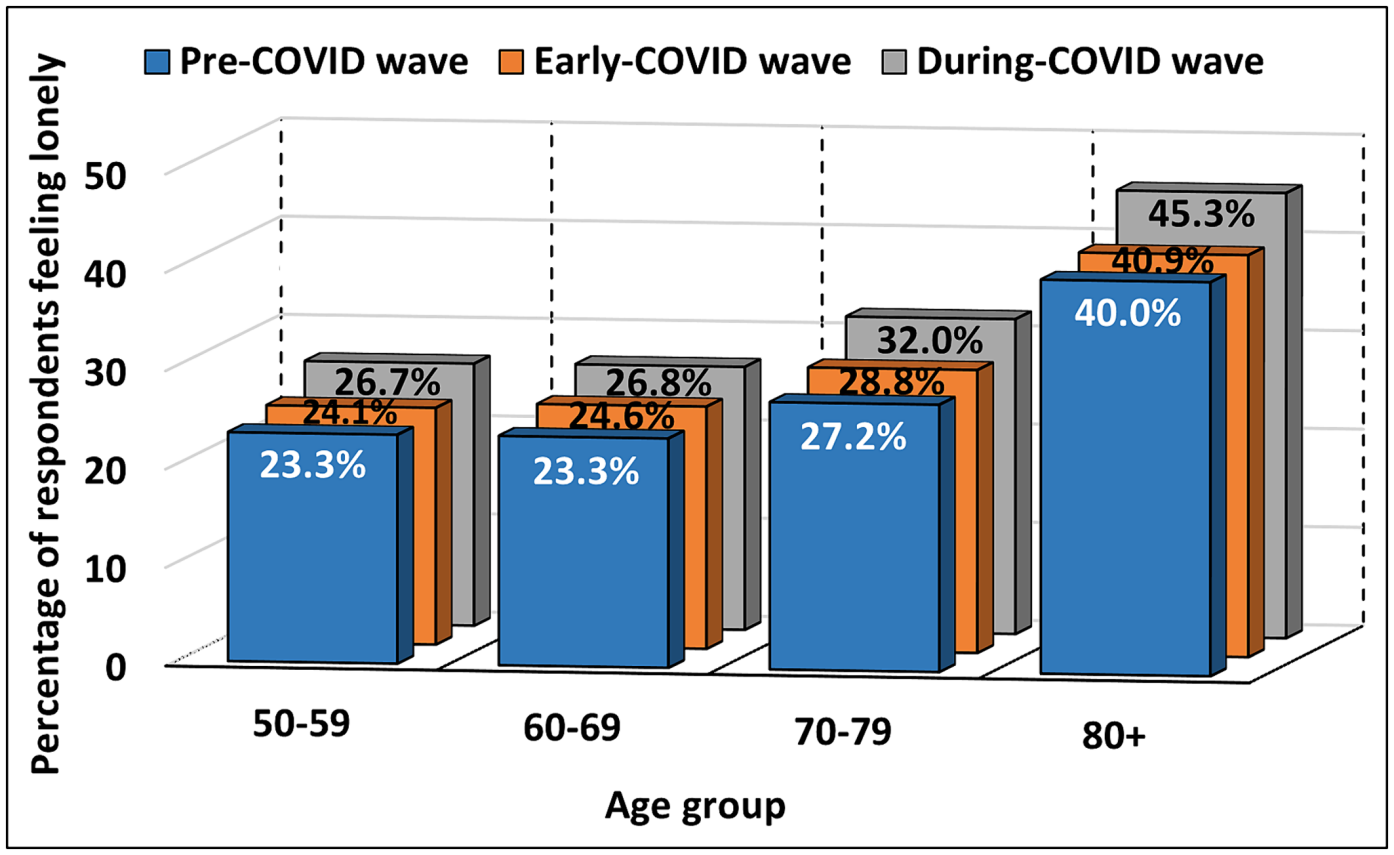
Percentage of respondents feeling lonely by age group in PCW, ECW and DCW.

A significant difference was found among all age groups for females (*p* < 0.001) and in the 60–80+ age groups for males (from *p* = 0.001 to *p* < 0.001). The mean rank scores of feeling lonely increased throughout the waves among ages 50–80+ for females and 80+ for males. The mean rank decreased from PCW to ECW and then increased in DCW (above the PCW values) among ages 60–69 for males, and the mean rank remained flat between PCW and ECW and then increased in DCW among ages 70–79 for males. A significant difference in feeling lonely scores was observed between PCW and ECW among ages 60–79 for females (*p* < 0.001), between PCW and DCW among ages 50–80+ for females (*p* < 0.001) and 70–80+ for males (*p* = 0.001), and between ECW and DCW among all age groups for both genders [from *p* = 0.015 to *p* < 0.001; subsection 2.3.2 in the Supplementary material: Changes in feeling lonely across three waves (PCW, ECW and DCW)]. Changes in the percentage of respondents who reported feeling lonely (“*some of the time*” and “*often*”) in each age group across the three waves (pre-COVID, early-COVID and during-COVID) are shown in Figure 5 and detailed in Table 4.

**Table 4:** Number and percentage of respondents feeling lonely by age group in PCW, ECW and DCW.

Age group	*N* (% age group)			
	Pre-COVID wave	Early-COVID wave	During-COVID wave	Total age group
50–59	1,002 (23.3%)	1,037 (24.1%)	1,148 (26.7%)	4,305 (100%)
60–69	2,737 (23.3%)	2,892 (24.6%)	3,141 (26.8%)	11,739 (100%)
70–79	2,790 (27.2%)	2,955 (28.8%)	3,289 (32.0%)	10,267 (100%)
80+	1,855 (40.0%)	1,894 (40.9%)	2,098 (45.3%)	4,632 (100%)

A combined analysis of face-to-face and electronic contacts revealed a significant difference in the face-to-face and electronic contact scores across the three waves (*p* < 0.001). The mean rank of the combined face-to-face and electronic contact scores decreased from 2.54 in PCW to 1.72 in ECW and increased to 1.74 in DCW. There was a significant difference in the combined face-to-face and electronic contact scores between PCW and ECW (*p* < 0.001), between PCW and DCW (*p* < 0.001), and between ECW and DCW (*p* = 0.002). The average face-to-face and electronic contacts score reported in DCW was higher by 0.5% compared to that of ECW, which in its turn was lower by 16.7% compared to PCW.

A significant difference was found among all age groups for both genders (*p* < 0.001). The mean rank of the combined face-to-face and electronic contact scores decreased from PCW to ECW and then increased in DCW (but being still below the PCW values) among ages 50–69 for both genders, 70–79 for males, and 80+ for females. The mean rank decreased from PCW to ECW and remained flat in DCW among ages 70–79 for females and 80+ for males. There was a significant difference in the combined face-to-face and electronic contact scores for all age groups for both genders between PCW and ECW (*p* < 0.001) and between PCW and DCW (*p* < 0.001). However, the combined face-to-face and electronic contact scores in ECW were not significantly different from those in DCW [subsection 2.3.3 in the Supplementary material: Changes in face-to-face and electronic communication (combined) across three waves (PCW, ECW and DCW)].

A separate analysis of the data on social integration differentiated between the subjects’ electronic and face-to-face contacts. For each respondent, the sum-scores for electronic contacts were higher than those for face-to-face contacts in ECW (by 28.6%) and DCW (by 13.1%). The subjects’ face-to-face contact scores in DCW (mean rank = 13,737.51) were significantly higher than in ECW (mean rank = 10,230.81, *p* < 0.001). This applies to all age groups for both genders [*p* < 0.001; subsection 2.3.4 in the Supplementary material: Changes in face-to-face communication across two waves (ECW and DCW)]. The average face-to-face contact score reported in DCW was higher compared to that of ECW by 19.8%. As for the subjects’ electronic contact scores, they were significantly lower in DCW (mean rank = 11,970.86) than in ECW (mean rank = 12,478.04, *p* < 0.001). This applies to ages 50–59 and 70 and above for both genders, and 60–69 for males [from *p* = 0.023 to *p* < 0.001; subsection 2.3.5 in the Supplementary material: Changes in electronic communication across two waves (ECW and DCW)]. The average electronic contact score reported in DCW was lower compared to that of ECW by 1.5%.

Given, on the one hand, a reported increase in electronic communication caused by pandemic-related restrictions on mobility (30), and on the other hand, a decrease in sadness or depression observed in our study across the three COVID-19 waves, we decided to check for possible relationships between (1) electronic communication, and (2) sadness or depression, for each of the waves for which the SHARE included a question about electronic communication—ECW and DCW. The analysis of relationships between having / not having electronic contacts and feeling / not feeling sad or depressed showed a significant association between these two parameters. The odds for sadness or depression were 21% lower (95% CI = 0.65–0.95) for individuals who reported having electronic contacts in ECW (*p* = 0.013) and 37% lower (95% CI = 0.54–0.73) for individuals who had electronic contacts in DCW (*p* < 0.001), compared to individuals who reported having no electronic contacts (Table 5).

**Table 5:** Association between electronic communication and sadness or depression (in ECW and DCW).

Wave	Odds ratio	95% Confidence Interval	Significance (*p*-value)	Pearson Chi-square
ECW	0.79	0.65–0.95	*p* = 0.013*	χ^2^(1) = 6.207
DCW	0.63	0.54–0.73	*p* < 0.001*	χ^2^(1) = 36.177

*Significant.

## 4 Discussion

This study examined longitudinal changes in physical, mental, and social parameters of adults aged 50 and older [from 26 EU countries (excluding Ireland), Israel, and Switzerland] before and during the COVID-19 pandemic.

### 4.1 Physical health parameters

Self-rated health is a reliable indicator of objective health and a predictor of mortality among older adults (55, 56). As expected, individuals rated their health as poorer during COVID-19 than before COVID-19 due to the adverse impact of the pandemic (57). This deterioration proved to be significant for females of all ages, and for males in all age groups except 60–69.

One of possible explanations for this can be physical inactivity forced on the population during COVID-19 lockdowns, social distancing requirements, and other restrictions (58). It is a known fact that exercising is associated with better self-rated health under normal social circumstances (59).

At the same time—contrary to our findings—some studies report improvements in self-rated health during the COVID-19 pandemic (60–62). However, none of the studies above views this result as objective. Peters et al. (62) links this reported improvement to subjective changes in health consciousness rather than objectively better health. The OECD (61) explains the improvements in self-rated health during the pandemic by the subjects’ change of perspective. Health issues that used to seem more serious were downplayed in the context of COVID-19. A similar explanation is found in the study by Kivi et al. (60) that attributes these positive changes to the contrast effect (63).

The number of illnesses or health conditions in DCW increased compared to PCW for both genders in all age groups. Among all illnesses and health conditions, the biggest increases were registered for heart attack or other heart problems, and high blood pressure or hypertension (4.9 and 4.1%, respectively). One possible explanation for an increase in heart problems could be a marked decline in heart failure hospitalizations during the COVID-19 pandemic. This was caused by fear of contracting the coronavirus, which caused patients to avoid visiting healthcare units, thus delaying treatment. After a delay, hospitalized patients had more severe symptoms at admission (64, 65). Another contributing factor could be stress, which has been linked to the development of cardiovascular diseases (66, 67). COVID-19 can be viewed as an extremely powerful factor causing increased stress in the population (68). As a result, researchers register a COVID-19-related increase in stress-induced cardiomyopathy presenting as acute heart failure (69). In addition, pandemic-related stress is viewed as one of possible reasons for significantly higher levels of blood pressure registered during COVID-19 (70).

The increase registered for diabetes or high blood sugar in DCW compared to PCW constituted 2.6%. Patients with diabetes are clearly a high-risk group for several reasons. First, diabetes has been identified as a risk factor for mortality from COVID-19 infection (71). Second, diabetes has been associated with a faster development of acute respiratory distress syndrome in hospitalized patients with COVID-19 (72). For these reasons, diabetic patients, particularly those with comorbidities, were urged to comply with social isolation and other measures aimed at preventing the spread of COVID-19 infection (16). As a result, they stayed indoors, had limited physical activity, postponed medical appointments, and failed to obtain required diabetes medications and supplies in time—all of which led to a deterioration in their condition (73–75).

A considerably lower increase in DCW compared to PCW was registered for chronic lung disease: 0.9%. Since the SARS-CoV-2 virus that caused COVID-19 affects the upper and lower respiratory tracts (76), individuals with chronic lung diseases such as chronic obstructive pulmonary disease (COPD) had worse health outcomes of the infection (77). Therefore, individuals with these conditions stayed at home to minimize the risk of infection, which resulted in reduced outdoor activity. Being devoid of sufficient physical activity had a negative impact on their health, as workout has been found to improve pulmonary functions (such as lung volumes, capacities, and flow rates) among healthy adults (78). This is in line with the recommendation by the European Respiratory Society (ERS) to engage symptomatic patients with chronic respiratory disease in pulmonary rehabilitation (79). The latter includes physical fitness aimed at alleviating the symptoms of the disease (80).

The same increase (0.9%) in DCW compared to PCW was registered for hip or femoral fractures. Similar conclusions about an increased number of falls and admitted hip fractures during the pandemic have been reported in other studies. One reason for that significant increase in hip fractures during the pandemic could be reduced support obtained by the older population from relatives and caregivers during the lockdown and traffic restrictions (81).

A slightly lower increase in DCW compared to PCW was registered for cancer or malignant tumor: 0.7%. The pandemic may have contributed to an increase in smoking-associated respiratory cancer (82). Increased pandemic-related smoking was associated with stress, boredom, isolation, economic crisis, and unemployment (82, 83). Another explanation of increased smoking during the pandemic, could be found in the so-called “smoker’s paradox” (or the nicotine hypothesis) that suggests smokers are protected from infection and severe complications of COVID-19 (84, 85). In addition, lung cancer and COVID-19 infection display similar symptoms, such as persistent cough, low oxygen levels, and breathlessness, which might have further delayed cancer diagnosis (82). As a result, patients (especially individuals aged over 50) were less likely to report potential cancer indicators during the pandemic compared to the pre-pandemic period. This decreased reporting is particularly evident for more common symptoms such as fever and coughs, and other alarming symptoms (86). Besides, aging patients with potential cancer symptoms have been reported to be unwilling to seek professional help. This was a particularly strong tendency in the first 6 months of the pandemic when the population was alarmed by media reports and concerned about catching an infection or infecting others (87).

### 4.2 Mental health and social parameters

Previous studies have established linkages and potential pathways between physical and mental health (88). Looking into such mental health parameters as sadness or depression and trouble sleeping, we discovered that, first, the number of individuals who reported these mental health issues in ECW was lower compared to PCW. Then, at a later stage of COVID-19 (DCW), there was an opposite tendency—with more individuals reporting sadness or depression and trouble sleeping compared to ECW. However, these increased numbers registered in the DCW (the end of the observation period) were still below the PCW values (the start of the observation period). This tendency was observed in both males and females of all ages.

Similar findings were reported in a study conducted in England between March and August 2020—the period we identify as PCW and ECW. Having surveyed over 70,000 adults, its authors found a peak in March 2020, followed by a gradual decrease in depressive symptoms till August 2020. The fastest decline in depression was registered across the strict lockdown period (25). The study suggested that the highest levels of depression and anxiety that occurred in the early stages of the lockdown might have been caused by individuals being psychologically affected even before the start of the lockdown. At that stage, there were reports of individuals self-isolating voluntarily prior to the announcement of the lockdown. Later, individuals may have adapted to the new reality, which can explain a rapid decline in their levels of depression and anxiety (25). Another explanation for a decrease in anxiety and depression observed during the COVID-19 pandemic could be age-related. A study analyzing the moderating effect of age on mental health found that older age (50+) was associated with lower levels of anxiety and depression compared to younger age (18-49 years). Its authors concluded that older age may buffer against the negative impact of the COVID-19 pandemic on mental health (89). At the same time, several studies report a pandemic-related deterioration in mental health among younger populations (90, 91). Blanchflower and Bryson (90), for example, refer to the 20–24 age group as the one with the highest levels of anxiety, depression, and worry during the COVID-19 pandemic. The researchers also conclude that the levels of these negative emotional states decline with age, in line with our findings (90).

Studying the adult population of 50 and above, we registered the highest number of individuals reporting sadness or depression, and sleep problems in the pre-COVID wave as compared to both early-COVID and during-COVID waves. To examine whether this was an isolated peak triggered by pandemic-induced anxiety and uncertainty, or whether it represented a typical baseline, we included in the analysis two additional SHARE waves—4 years pre-COVID and 2 years pre-COVID. A longitudinal analysis for each of them as compared to pre-COVID, early-COVID, and during-COVID waves showed that the peak observed in the pre-COVID wave was a typical baseline which we have found to go down during the pandemic. These findings suggest that it was not an isolated peak caused by the pandemic-induced uncertainty and stress. Therefore, there had to be another factor that had a positive influence on the individuals’ mental health during the COVID-19 outbreak.

Based on the results from the SHARE database, this positive impact can be attributed to electronic communication. As shown above, individuals had more electronic contact in ECW and less in DCW. Respectively, the number of cases involving sadness or depression, and sleep problems, was lower in ECW and higher in DCW. Additionally, the results indicated that having electronic contact might reduce the odds of experiencing sadness or depression. In our study, although face-to-face contact increased while electronic contact decreased for the analyzed waves (ECW and DCW), the frequency scores of electronic contacts proved to be higher than those of face-to-face contacts.

Following the social distancing rules introduced to prevent the spread of the coronavirus, electronic communication very much replaced face-to-face communication (92). Overall, before the COVID-19 outbreak, electronic communication was available but was not used as much as it was during the pandemic (30). During the pandemic, it promoted the perception of social support, reduced the psychological effects of closures, mitigated negative emotions, increased the sense of belonging, and buffered the adverse mental health effects of depression during lockdown periods (93, 94). Fancourt et al. (25) explained that the extensive use of virtual and digital communication during the COVID-19 pandemic might have helped to ease the burden of the lockdown itself and reduced the fear of missing out, which is associated with depression (95). In particular, electronic communication with a friend was found to decrease loneliness, anxiety, and depressive symptoms during the pandemic (96).

Reducing the levels of depression was accompanied by a reduction in sleeping problems. After all, patients with depression often have difficulty falling asleep and have frequent nighttime and early morning awakenings (97, 98). Overall, patients with mood disorders exhibit higher rates of sleep disturbance than the general population (99). This suggests a need to maintain electronic social networks, especially for adults aged 50 and above, to mitigate mental health problems caused by social distancing.

For humans as social beings, physical distancing affected social life, which is crucial for our evolution and survival (100). Loneliness, viewed as an individual’s subjective perception of lack of social connections and relationships, increased throughout the waves under analysis. Other studies also found a gradual increase in loneliness from the pre-pandemic period to the COVID-19 period among individuals aged 50 and above (101–103). Based on the results we achieved, throughout the pandemic, the mean rank scores for feeling lonely in females increased gradually and were significant across all age groups – which was not the case with males. These results are in line with those of Savage et al. (104) who conducted a cross-sectional study among older adults in Canada during the pandemic. The authors found that women had increased odds of loneliness compared to men, whether living alone or with others. Regardless of the pandemic, Pyle and Evans (105) found that women were significantly more likely than men to report feeling lonely. The article hypothesized that this difference may be rooted in how men and women reflect on their personal experiences of loneliness or that men are more reluctant than women to report undesirable social experiences, such as loneliness (105).

Lockdowns and social distancing rules also had an effect on the employment situation. The number of individuals working decreased throughout the waves, primarily between PCW and DCW. The pandemic drastically changed employment status globally, especially for the older ages. Bui et al. (106) found that unemployment rates increased to 15.4% for people aged 65 years and older compared to 13.0% for those aged 25–44. Our results show that the number of working individuals changed over time for ages 50–79 for both genders and males aged 80+. The gradual change in the employment situation was not uniform between age groups. In the 60–69 age group, both males and females aged 60–69 worked less throughout the three tested waves, while the 50–59 age group demonstrated a gradual decline in working cases between PCW (conducted between October 2019 and March 2020) and ECW (conducted between June–September 2020) and an increase between ECW and DCW (conducted between June–August 2021). Goda et al. (107) also found that employment among older workers declined sharply in April 2020 before slowly recovering and leveling off. The researchers explained that for ages 50–61, most of the decline (about 63%) was due to increased unemployment, while the rest was due to increases in labor force exits for reasons other than retirement and disability. In addition, they found that in the 62–70 age group, 50% of the decline was attributed to increased unemployment and 30%—to retirement (107). As for the age 70–79 for both genders and age 80+ for males, we registered an increase in the number of working individuals from PCW to ECW and then a decrease in DCW, with even fewer working cases than in PCW.

We find it important to stress that our study was conducted on a sample population from EU countries, Israel, and Switzerland. Considering diverse levels of economic development in other regions across the world, our findings and conclusions may not apply to a broader context, particularly to developing countries with a very different income level and technological development.

### 4.3 Limitation

One of the limitations of the current study is changes in how data were collected by the SHARE before and during the COVID-19 pandemic. To respond to the COVID-19 crisis, the Survey of Health, Ageing and Retirement in Europe adapted its data collection mode, questionnaire content, sample design, and actual fieldwork (43). These adaptations could potentially have an impact on the results we obtained and the conclusions we drew. At the same time, the changes above do not prevent SHARE from reaching its objective—collecting internationally comparable data in the health sector, social sector, and economic sector (43)—which means our results are still valid and reliable.

Another limitation could be that some respondents may have interpreted video conference calls (like Zoom, Skype, WhatsApp video calls, etc.) as instances of face-to-face rather than electronic communication. An interpretation like this could result in higher scores for face-to-face contacts and lower scores for electronic contacts, thus potentially interfering with the results we obtained for the early-COVID and during-COVID waves (June–September 2020 and June–August 2021, respectively). However, the decrease in face-to-face communication scores we registered in the ECW is in line with the lockdown introduced in Europe on 18 March 2020, which affected more than 250 million people (108). As for the increase in face-to-face communication scores that we registered in the DCW, it chronologically coincides with the vaccination campaign started by the European Commission in late December 2020 (109), which resulted in more frequent face-to-face interactions (110).

## 5 Conclusion

The COVID-19 pandemic affected all aspects of human life—physical health, mental health and social networks worldwide. This study sought to conduct a quantitative analysis of the COVID-19 impact on the physical, mental, and social parameters in adults aged 50 and older. One of its major (and unexpected) findings is a significant improvement in mental health between the pre-COVID and during-COVID periods. We tend to attribute this improvement to a more active use of electronic means of communication during COVID-19. Our study also confirmed a deterioration in the individuals’ subjective health status and a bigger number of illnesses or health conditions in DCW compared to PCW, especially for cardiovascular diseases. As for the effect of the COVID-19 pandemic on social parameters, the key findings are vastly lower employment rates and dramatically higher rates of loneliness from pre-COVID to early- to during-COVID waves, as well as significantly lower scores of social contacts from pre-COVID to during-COVID waves.

The results obtained highlight the importance of electronic contact as an effective form of social interaction that can contribute to the mental health of adults. Therefore, it seems crucial to make internet access more available to older people, provide them with personal computers, and develop their computer skills. This approach should be used for developing better solutions for public welfare policy and viewed as a public health priority. Besides, it seems important to educate the population of all age groups about the importance of social, particularly electronic contacts, and their impact on mental health. A practical step in this direction would be bridging the generation gap and encouraging young adults to make technology more available to their older relatives, thus protecting them from potential mental health risks associated with pandemics and other global or personal crises.

Further studies are needed to examine (1) the use of electronic communications between during-COVID and post-COVID periods and (2) its correlation with the rates of depression, sadness, and sleeping problems. At this stage, it can be predicted that a decreased use of electronic communications may contribute to a return to the pre-COVID high baseline values of depression, sadness, and sleeping problems.

## Data availability statement

The original contributions presented in the study are included in the article/Supplementary material, further inquiries can be directed to the corresponding author. SHARE data is free of charge for scientific use globally (available at www.share-eric.eu/data/data-access).

## Ethics statement

Ethical approval and participant consent were not required for this study since it was based solely on secondary data analysis (see www.share-eric.eu).

## Author contributions

SM: Visualization, Methodology, Investigation, Formal analysis, Data curation, Conceptualization, Writing – review & editing, Writing – original draft. IR: Writing – review & editing, Investigation. RM: Writing – review & editing, Supervision, Project administration, Methodology, Conceptualization.
