# Supplementary material for: Improvements in mental health associated with increased electronic communication and deterioration in physical health in adults aged 50+ during the COVID-19 pandemic
Source: Front Public Health. 2024 Jun 21;12:1369707. doi: 10.3389/fpubh.2024.1369707 (PMC11224488; doi:10.3389/fpubh.2024.1369707)
Supplement: Supplementary file 1 [file Data_Sheet_1.DOCX]

Supplementary Material

# SHARE questions used for the paper: Improvements in mental health associated with increased electronic communication and deterioration in physical health in adults aged 50+ during the COVID-19 pandemic

The questions used for the paper were taken from the SHARE (Survey of Health, Ageing and Retirement in Europe) Waves 6, 7, 8, 8 Corona Survey 1 and 9 Corona Survey 2 (DOIs: 10.6103/SHARE.w6.800, 10.6103/SHARE.w7.800, 10.6103/SHARE.w8.800, 10.6103/SHARE.w8ca.800 and 10.6103/SHARE.w9ca800).

SHARE questionnaires are available online:

Wave 6: <https://share-eric.eu/fileadmin/user_upload/Questionnaires/Q-Wave_6/w6_en_capi_main-Generic.pdf>

Wave 7: <https://share-eric.eu/fileadmin/user_upload/Questionnaires/Q-Wave_7/w7_en_capi_main-Generic.pdf>

Wave 8: <https://share-eric.eu/fileadmin/user_upload/Questionnaires/Q-Wave_8/paperverstion_en_GB_8_2_5b.pdf>

Wave 8 Corona Survey 1: <https://share-eric.eu/fileadmin/user_upload/Questionnaires/Corona_Questionnaire_1/corona_en_cati-Generic.pdf>

Wave 9 Corona Survey 2: <https://share-eric.eu/fileadmin/user_upload/Questionnaires/Corona_Questionnaire_2/corona2_en_cati-Generic.pdf>

The letters and numbers combination in the parentheses is the question code used by the SHARE questionnaires.

## Physical health parameters-related questions:

Wave 8 (PCW)

HEALTH IN GENERAL QUESTION 2 (PH003_HealthGen2)

Question

Would you say your health is...

Read out.;

Response options:

1. Excellent

2. Very good

3. Good

4. Fair

5. Poor

Wave 9 Corona Survey 2 (DCW)

HEALTH: RATING OF SUBJECTIVE HEALTH (CAPH003_)

Question

Would you say your health is excellent, very good, good, fair, or poor?

Response options:

1. Excellent

2. Very good

3. Good

4. Fair

5. Poor

Wave 8 (PCW)

DOCTOR TOLD YOU HAD CONDITIONS (PH006_DocCond)

Question

Please look at card 7. *[Has a doctor ever told you that you had/ Do you currently have]* any of the conditions on this card? *[With this we mean that a doctor has told you that you have this condition, and that you are either currently being treated for or bothered by this condition.]*

Please tell me the number or numbers of the conditions.

Code all that apply.;

Response options:

1. A heart attack including myocardial infarction or coronary thrombosis or any other heart problem including congestive heart failure (ph006d1)

2. High blood pressure or hypertension (ph006d2)

5. Diabetes or high blood sugar (ph006d5)

6. Chronic lung disease such as chronic bronchitis or emphysema (ph006d6)

10. Cancer or malignant tumour, including leukaemia or lymphoma, but excluding minor skin cancers (ph006d10)

14. Hip fracture (ph006d14)

Wave 9 Corona Survey 2 (DCW)

ILLNESSES OR HEALTH CONDITIONS (CAH004_)

Question

Do you have any of the following illnesses or health conditions? Please answer yes or no to each category:

*IWER: With this we mean that a doctor has told you that you have this condition, and that you are either currently being treated for or bothered by this condition.*

*IWER: READ OUT.*

Hip fracture? (CAH004_1)

Diabetes or high blood sugar? (CAH004_2)

High blood pressure or hypertension? (CAH004_3)

A heart attack including myocardial infarction or coronary thrombosis or any other heart problem including congestive heart failure? (CAH004_4)

Chronic lung disease such as chronic bronchitis or emphysema? (CAH004_5)

Cancer or malignant tumour, including leukaemia or lymphoma, but excluding minor skin cancers? (CAH004_6)

Response options:

1. Yes

5. No

-1. Don't know

-2. Refusal

## Mental health parameters-related questions (2019-2021):

Waves 8, 8 Corona Survey 1 and 9 Corona Survey 2 (PCW, ECW and DCW)

DEPRESSION (Wave 8: MH002_Depresion, Corona surveys: CAMH002_)

Question

*In the last month, have you been sad or depressed?*

*IWER: If participant asks for clarification, say 'by sad or depressed, we mean miserable, in low spirits, or blue'*

Response options:

1. Yes

5. No

Waves 8, 8 Corona Survey 1 and 9 Corona Survey 2 (PCW, ECW and DCW)

TROUBLE SLEEPING (Wave 8: MH007_Sleep, Corona surveys: CAMH007_)

Question

*Have you had trouble sleeping recently?*

(Corona surveys: *IWER: DO NOT READ OUT)*

Response options:

1. Trouble with sleep or recent change in pattern

2. No trouble sleeping

## Social parameters-related questions:

Wave 8 (PCW)

CURRENT JOB SITUATION (EP005_CurrentJobSit)

Question

Please look at card 21. In general, which of the following best describes your current employment situation?

Code only one

Only if R in doubt then refer to the following:

1. Retired (retired from own work, including semi-retired, partially retired, early retired, pre-retired). Retired refers to retired from own work only. Recipients of survivor pensions who do not receive pensions from own work should not be coded as retired. If they do not fit in categories 2 through 5, they should go into other.

Response options:

1. Retired

2. Employed or self-employed (including working for family business)

3. Unemployed

4. Permanently sick or disabled

5. Homemaker

97. Other

Wave 8 Corona Survey 1 (ECW)

WORK: UNEMPLOYED, LAID OFF OR BUSINESS CLOSED DUE TO COVID-19 (CAW002_)

Question

Due to the Corona crisis have you become unemployed, were laid off or had to close your business?

*IWER: Business closure can be both temporarily or permanently.*

Response options:

1. Yes

5. No

Wave 9 Corona Survey 2 (DCW)

WORK: CURRENT EMPLOYMENT SITUATION (CAEP005_CurrentJobSit)

Question

Which of the following best describes your **current** employment situation?

*Code only one. IWER: Only if R is in doubt then refer to the following: 1. Retired (retired from own work, including semi-retired, partially retired, early retired, pre-retired). Retired refers to retired from own work only. Recipients of survivor pensions who do not receive pensions from own work should not be coded as retired. If they do not fit in categories 2 through 5, they should go into other.*

*IWER: Read out*

Response options:

1. Retired

2. Employed or self-employed (including working for family business)

3. Unemployed

4. Permanently sick or disabled

5. Homemaker

97. Other

Wave 8 (PCW)

NETWORK CONTACT (FACE-TO-FACE & ELECTRONIC) (SN007_NetworkContact)

Question

During the past twelve months, how often did you have contact (average) with*;*

Spouse (spouse_contact)

Family members (fam_contact)

Children (child_contact)

Siblings (sibling_contact)

Parents (parent_contact)

Friends (friend_contact)

Formal helpers (formal_contact)

Others (other_contact)

in social network; either in person, by phone or mail, email or any other electronic means?

Response options:

1. Daily

2. Several times a week

3. About once a week

4. About every two weeks

5. About once a month

6. Less than once a month

7. Never

Wave 8 Corona Survey 1 (ECW)

SOCIAL NETWORKS (FACE-TO-FACE) (CAS003_)

Question

Since the outbreak of Corona, how often did you have personal contact, that is, face to face, with the following people from outside your home? Was it daily, several times a week, about once a week, less often, or never?

*IWER: Read out each relationship and check the appropriate answer.*

Own children (CAS003_1)

Own parents (CAS003_2)

Other relatives (CAS003_3)

Other non-relatives like neighbors, friends, or colleagues (CAS003_4)

Response options:

1. Daily

2. Several times a week

3. About once a week

4. Less often

5. Never

99. Not applicable

-1. Don't know

-2. Refusal

Wave 9 Corona Survey 2 (DCW)

SOCIAL NETWORKS (FACE-TO-FACE) (CAS103_)

Question

During the last three months, how often did you have personal contact, that is, face to face, with the following people from outside your home? Was it daily, several times a week, about once a week, less often, or never?

*IWER: Read out each relationship and check the appropriate answer.*

*If respondent does not have any living parents, children, grandchildren or relatives, code 'Not applicable'.*

Own children (CAS103_1)

Own parents (CAS103_2)

Other relatives (CAS103_3)

Other non-relatives like neighbors, friends, or colleagues (CAS103_4)

Response options:

1. Daily

2. Several times a week

3. About once a week

4. Less often

5. Never

99. Not applicable

-1. Don't know

-2. Refusal

Wave 8 Corona Survey 1 (ECW)

SOCIAL NETWORKS (ELECTRONIC) (CAS004_)

Question

Since the outbreak of Corona, how often did you have contact by phone, email or any other electronic means with the following people from outside your home? (Was it daily, several times a week, about once a week, less often, or never?)

*IWER: Read out each relationship and check the appropriate answer.*

Own children (CAS004_1)

Own parents (CAS004_2)

Other relatives (CAS004_3)

Other non-relatives like neighbors, friends, or colleagues (CAS004_4)

Response options:

1. Daily

2. Several times a week

3. About once a week

4. Less often

5. Never

99. Not applicable

-1. Don't know

-2. Refusal

Wave 9 Corona Survey 2 (DCW)

SOCIAL NETWORKS (ELECTRONIC) (CAS104_)

Question

During the last three months, how often did you have contact by phone, email or any other electronic means with the following people from outside your home? (Was it daily, several times a week, about once a week, less often, or never?)

*IWER: Read out each relationship and check the appropriate answer.*

Own children (CAS104_1)

Own parents (CAS104_2)

Other relatives (CAS104_3)

Other non-relatives like neighbours, friends, or colleagues (CAS104_4)

Response options:

1. Daily

2. Several times a week

3. About once a week

4. Less often

5. Never

-1. Don't know

-2. Refusal

Waves 8, 8 Corona Survey 1 and 9 Corona Survey 2 (PCW, ECW and DCW)

HOW OFTEN LONELY (Wave 8: MH037_lonely, Corona surveys: CAMH037_)

Question

How much of the time do you feel lonely?

(Wave 8: Repeat if necessary, Corona waves: …Often, some of the time, or hardly ever or never?)

Response options:

1. Often

2. Some of the time

3. Hardly ever or never

## Mental health parameters-related questions (2015, 2017):

Waves 6 and 7 (4yPCW and 2yPCW)

DEPRESSION (MH002_Depresion)

Question

*In the last month, have you been sad or depressed?*

*IWER: If participant asks for clarification, say 'by sad or depressed, we mean miserable, in low spirits, or blue'*

Response options:

1. Yes

5. No

Waves 6 and 7 (4yPCW and 2yPCW)

TROUBLE SLEEPING (MH007_Sleep)

Question

*Have you had trouble sleeping recently?*

(Corona surveys: *IWER: DO NOT READ OUT)*

Response options:

1. Trouble with sleep or recent change in pattern

2. No trouble sleeping

# Gender specific and age specific changes in physical health, mental health and social parameters across the SHARE waves

## Changes in physical health parameters across two waves (PCW and DCW):

### Changes in rating of subjective health

| **Age group** | **Gender** | **Wilcoxon Signed-Rank test** |
| --- | --- | --- |
| 50-80+ | Males and females  N = 31,461 | Z = -9.987, *p* < 0.001*  Mean rank: PCW = 7,475.27, DCW = 7,350.93 |
| 50-59 | Males  N = 1,562 | Z = -1.990, *p* = 0.047*  Mean rank: PCW = 379.73, DCW = 379.23 |
|  | Females  N = 2,778 | Z = -3.391, *p* = 0.001*  Mean rank: PCW = 661.46, DCW = 652.73 |
| 60-69 | Males  N = 5,035 | Z = -1.834, *p* = 0.067  Mean rank: PCW = 1,202.42, DCW = 1,201.55 |
|  | Females  N = 6,817 | Z = -3.915, *p* < 0.001*  Mean rank: PCW = 1,625.58, DCW = 1,590.21 |
| 70-79 | Males  N = 4,492 | Z = -4.929, *p* < 0.001*  Mean rank: PCW = 1,092.06, DCW = 1,058.70 |
|  | Females  N = 5,917 | Z = -3.961, *p* < 0.001*  Mean rank: PCW = 1,346.67, DCW = 1,335.47 |
| 80+ | Males  N = 1,886 | Z = -5.569, *p* < 0.001*  Mean rank: PCW = 477.74, DCW = 464.96 |
|  | Females  N = 2,974 | Z = -3.331, *p* = 0.001*  Mean rank: PCW = 692.99, DCW = 670.20 |

* Significant

### Changes in the number of illnesses or health conditions

| **Age group** | **Gender** | **Wilcoxon Signed-Rank test** |
| --- | --- | --- |
| 50-80+ | Males and females  N = 31,216 | Z = -32.091, *p* < 0.001*  Mean rank: PCW = 5,495.61, DCW = 5,770.91 |
| 50-59 | Males  N = 1,553 | Z = -8.860, *p* < 0.001*  Mean rank: PCW = 225.11, DCW = 239.12 |
|  | Females  N = 2,761 | Z = -9.513, *p* < 0.001*  Mean rank: PCW = 329.30, DCW = 348.72 |
| 60-69 | Males  N = 4,994 | Z = -13.759, *p* < 0.001*  Mean rank: PCW = 834.97, DCW = 876.31 |
|  | Females  N = 6,776 | Z = -15.063, *p* < 0.001*  Mean rank: PCW = 983.72, DCW = 1,023.02 |
| 70-79 | Males  N = 4,468 | Z = -12.430, *p* < 0.001*  Mean rank: PCW = 926.92, DCW = 970.64 |
|  | Females  N = 5,880 | Z = -13.223, *p* < 0.001*  Mean rank: PCW = 1,110.44, DCW = 1,180.96 |
| 80+ | Males  N = 1,868 | Z = -7.701, *p* < 0.001*  Mean rank: PCW = 410.02, DCW = 450.29 |
|  | Females  N = 2,916 | Z = -9.118, *p* < 0.001*  Mean rank: PCW = 672.26, DCW = 689.38 |

* Significant

### Changes in the type of illness or health condition**

| **Age group** | 50-80+ | 50-59 | | 60-69 | |
| --- | --- | --- | --- | --- | --- |
| **Gender** | Males and females  N = 31,216 | Males  N = 1,553 | Females  N = 2,761 | Males  N = 4,994 | Females  N = 6,776 |
| **High blood pressure or hypertension** | χ^2^ = 277.132,  *p* < 0.001* | χ^2^ = 35.088,  *p* < 0.001* | χ^2^ = 48.878,  *p* < 0.001* | χ^2^ = 80.330,  *p* < 0.001* | χ^2^ = 79.970,  *p* < 0.001* |
| **Diabetes or high blood sugar** | χ^2^ = 296.763,  *p* < 0.001* | χ^2^ = 27.313,  *p* < 0.001* | χ^2^ = 13.752,  *p* < 0.001* | χ^2^ = 51.124,  *p* < 0.001* | χ^2^ = 78.607,  *p* < 0.001* |
| **Heart attack or other heart problem** | χ^2^ = 567.436,  *p* < 0.001* | χ^2^ = 18.720,  *p* < 0.001* | χ^2^ = 28.547,  *p* < 0.001* | χ^2^ = 69.882,  *p* < 0.001* | χ^2^ = 96.488,  *p* < 0.001* |
| **Chronic lung disease** | χ^2^ = 49.842,  *p* < 0.001* | χ^2^ = 4.438,  *p* = 0.035* | χ^2^ = 13.913,  *p* < 0.001* | χ^2^ = 7.788,  *p* = 0.005* | χ^2^ = 16.550,  *p* < 0.001* |
| **Cancer or malignant tumor** | χ^2^ = 26.070,  *p* < 0.001* | χ^2^ = 3.692,  *p* = 0.055 | χ^2^ = 0.771,  *p* = 0.380 | χ^2^ = 8.708,  *p* = 0.003* | χ^2^ = 3.568,  *p* = 0.059 |
| **Hip fracture or femoral fracture** | χ^2^ = 77.970,  *p* < 0.001* | χ^2^ = 2.370,  *p* = 0.124 | χ^2^ = 3.115,  *p* = 0.078 | χ^2^ = 5.823,  *p* 0 =.016* | Z = 8.507,  *p* = 0.004* |

| **Age group** | 70-79 | | 80+ | |
| --- | --- | --- | --- | --- |
| **Gender** | Males  N = 4,468 | Females  N = 5,880 | Males  N = 1,868 | Females  N = 2,916 |
| **High blood pressure or hypertension** | χ^2^ = 32.504,  *p* < 0.001* | χ^2^ = 33.770,  *p* < 0.001* | χ^2^ = 5.470,  *p* = 0.019* | χ^2^ = 2.947,  *p* = 0.086 |
| **Diabetes or high blood sugar** | χ^2^ = 36.497,  *p* < 0.001* | χ^2^ = 40.096,  *p* < 0.001* | χ^2^ = 28.699,  *p* < 0.001* | χ^2^ = 26.294,  *p* < 0.001* |
| **Heart attack or other heart problem** | χ^2^ = 93.157,  *p* < 0.001* | χ^2^ = 135.551,  *p* < 0.001* | χ^2^ = 40.386,  *p* < 0.001* | χ^2^ = 84.039,  *p* < 0.001* |
| **Chronic lung disease** | χ^2^ = 15.062,  *p* < 0.001* | χ^2^ = 0.981,  *p* = 0.322 | χ^2^ = 4.500,  *p* = 0.034* | χ^2^ = 0.153,  *p* = 0.696 |
| **Cancer or malignant tumor** | χ^2^ = 6.717,  *p* = 0.010* | χ^2^ = 4.037,  *p* = 0.045* | χ^2^ = 0.221,  *p* = 0.638 | χ^2^ = 0.894,  *p* = 0.344 |
| **Hip fracture or femoral fracture** | χ^2^ = 8.463,  *p* = 0.004* | χ^2^ = 19.980,  *p* < 0.001* | χ^2^ = 4.129,  *p* = 0.042* | χ^2^ = 22.458,  *p* < 0.001* |

* Significant, ** McNemar test

## Changes in mental health parameters across three waves (PCW, ECW and DCW), and across four waves (2yPCW / 4yPCW, PCW, ECW and DCW):

### Changes in sadness or depression across three waves (PCW, ECW and DCW)

| **Age group** | **Gender** | **Cochran’s Q test and McNemar test as Post Hoc analysis** | | |
| --- | --- | --- | --- | --- |
| 50-80+ | Males and females  N = 30,936 | χ^2^(2) = 2,228.234, *p* < 0.001*  Freq.: PCW = 12,108 (39%), ECW = 7,663 (25%), DCW = 9,130 (30%). | | |
|  |  | PCW vs. ECW | PCW vs. DCW | ECW vs. DCW |
|  |  | χ^2^ = 1,994.258,  *p* < 0.001* | χ^2^ = 894.302,  *p* < 0.001* | χ^2^ = 275.074,  *p* < 0.001* |
| 50-59 | Males  N = 1,542 | χ^2^(2) = 153.534, *p* < 0.001*  Freq.: PCW = 488 (32%), ECW = 255 (17%), DCW = 305 (20%). | | |
|  |  | PCW vs. ECW | PCW vs. DCW | ECW vs. DCW |
|  |  | χ^2^ = 122.606,  *p* < 0.001* | χ^2^ = 76.147,  *p* < 0.001* | χ^2^ = 7.950,  *p* = 0.005* |
|  | Females  N = 2,761 | χ^2^(2) = 231.002, *p* < 0.001*  Freq.: PCW = 1,138 (41%), ECW = 711 (26%), DCW = 817 (30%). | | |
|  |  | PCW vs. ECW | PCW vs. DCW | ECW vs. DCW |
|  |  | χ^2^ = 197.042,  *p* < 0.001* | χ^2^ = 133.904,  *p* < 0.001* | χ^2^ = 14.739,  *p* < 0.001* |
| 60-69 | Males  N = 4,953 | χ^2^(2) = 318.198, *p* < 0.001*  Freq.: PCW = 1,357 (27%), ECW = 762 (15%), DCW = 923 (19%). | | |
|  |  | PCW vs. ECW | PCW vs. DCW | ECW vs. DCW |
|  |  | χ^2^ = 269.135,  *p* < 0.001* | χ^2^ = 142.686,  *p* < 0.001* | χ^2^ = 27.033,  *p* < 0.001* |
|  | Females  N = 6,767 | χ^2^(2) = 537.737, *p* < 0.001*  Freq.: PCW = 2,903 (43%), ECW = 1,861 (28%), DCW = 2,089 (31%). | | |
|  |  | PCW vs. ECW | PCW vs. DCW | ECW vs. DCW |
|  |  | χ^2^ = 452.288,  *p* < 0.001* | χ^2^ = 278.186,  *p* < 0.001* | χ^2^ = 26.782,  *p* < 0.001* |
| 70-79 | Males  N = 4,408 | χ^2^(2) = 240.772, *p* < 0.001*  Freq.: PCW = 1,248 (28%), ECW = 735 (17%), DCW = 999 (23%). | | |
|  |  | PCW vs. ECW | PCW vs. DCW | ECW vs. DCW |
|  |  | χ^2^ = 225.016,  *p* < 0.001* | χ^2^ = 51.296,  *p* < 0.001* | χ^2^ = 75.512,  *p* < 0.001* |
|  | Females  N = 5,850 | χ^2^(2) = 526.884, *p* < 0.001*  Freq.: PCW = 2,818 (48%), ECW = 1,833 (31%), DCW = 2,176 (37%). | | |
|  |  | PCW vs. ECW | PCW vs. DCW | ECW vs. DCW |
|  |  | χ^2^ = 475.334,  *p* < 0.001* | χ^2^ = 199.651,  *p* < 0.001* | χ^2^ = 73.148,  *p* < 0.001* |
| 80+ | Males  N = 1,804 | χ^2^(2) = 67.938, *p* < 0.001*  Freq.: PCW = 609 (34%), ECW = 419 (23%), DCW = 524 (29%). | | |
|  |  | PCW vs. ECW | PCW vs. DCW | ECW vs. DCW |
|  |  | χ^2^ = 62.889,  *p* < 0.001* | χ^2^ = 12.187,  *p* < 0.001* | χ^2^ = 23.876,  *p* < 0.001* |
|  | Females  N = 2,851 | χ^2^(2) = 209.342, *p* < 0.001*  Freq.: PCW = 1,547 (54%), ECW = 1,087 (38%), DCW = 1,297 (45%). | | |
|  |  | PCW vs. ECW | PCW vs. DCW | ECW vs. DCW |
|  |  | χ^2^ = 197.637,  *p* < 0.001* | χ^2^ = 59.049,  *p* < 0.001* | χ^2^ = 47.274,  *p* < 0.001* |

* Significant

### Changes in sadness or depression across four waves (2yPCW, PCW, ECW and DCW)

| **Age group** | **Gender** | **Cochran’s Q test and McNemar test as Post Hoc analysis** | | |
| --- | --- | --- | --- | --- |
| 50-80+ | Males and females  N = 5,715 | χ^2^(3) = 352.490, *p* < 0.001*  Freq.: 2yPCW = 2,052 (36%), PCW = 2,117 (37%), ECW = 1,475 (26%), DCW = 1,652 (29%). | | |
|  |  | 2yPCW vs. PCW | 2yPCW vs. ECW | 2yPCW vs. DCW |
|  |  | χ^2^ = 2.587,  *p* = 0.108 | χ^2^ = 182.395,  *p* < 0.001* | χ^2^ = 89.539,  *p* < 0.001* |
|  |  | PCW vs. ECW | PCW vs. DCW | ECW vs. DCW |
|  |  | χ^2^ = 250.232,  *p* < 0.001* | χ^2^ = 127.018,  *p* < 0.001* | χ^2^ = 23.308,  *p* < 0.001* |

* Significant

### Changes in sadness or depression across four waves (4yPCW, PCW, ECW and DCW)

| **Age group** | **Gender** | **Cochran’s Q test and McNemar test as Post Hoc analysis** | | |
| --- | --- | --- | --- | --- |
| 50-80+ | Males and females  N = 21,283 | χ^2^(3) = 2,501.632, *p* < 0.001*  Freq.: 4yPCW = 8,563 (40%), PCW = 8,280 (39%), ECW = 5,155 (24%), DCW = 6,067 (29%). | | |
|  |  | 4yPCW vs. PCW | 4yPCW vs. ECW | 4yPCW vs. DCW |
|  |  | χ^2^ = 11.815,  *p* = 0.001* | χ^2^ = 1,568.601,  *p* < 0.001* | χ^2^ = 849.949,  *p* < 0.001* |
|  |  | PCW vs. ECW | PCW vs. DCW | ECW vs. DCW |
|  |  | χ^2^ = 1,443.054,  *p* < 0.001* | χ^2^ = 731.054,  *p* < 0.001* | χ^2^ = 160.651,  *p* < 0.001* |

* Significant

### Changes in trouble sleeping or recent change in sleep pattern across three waves (PCW, ECW and DCW)

| **Age group** | **Gender** | **Cochran’s Q test and McNemar test as Post Hoc analysis** | | |
| --- | --- | --- | --- | --- |
| 50-80+ | Males and females  N = 31,046 | χ^2^(2) = 1,063.078, *p* < 0.001*  Freq.: PCW = 11,371 (37%), ECW = 8,396 (27%), DCW = 9,828 (32%). | | |
|  |  | PCW vs. ECW | PCW vs. DCW | ECW vs. DCW |
|  |  | χ^2^ = 1,020.030,  *p* < 0.001* | χ^2^ = 275.811,  *p* < 0.001* | χ^2^ = 266.081,  *p* < 0.001* |
| 50-59 | Males  N = 1,548 | χ^2^(2) = 37.639, *p* < 0.001*  Freq.: PCW = 387 (25%), ECW = 282 (18%), DCW = 368 (24%). | | |
|  |  | PCW vs. ECW | PCW vs. DCW | ECW vs. DCW |
|  |  | χ^2^ = 31.906,  *p* < 0.001* | χ^2^ = 0.873,  *p* = 0.350 | χ^2^ = 25.087,  *p* < 0.001* |
|  | Females  N = 2,761 | χ^2^(2) = 131.635, *p* < 0.001*  Freq.: PCW = 1,050 (38%), ECW = 738 (27%), DCW = 870 (32%). | | |
|  |  | PCW vs. ECW | PCW vs. DCW | ECW vs. DCW |
|  |  | χ^2^ = 125.286,  *p* < 0.001* | χ^2^ = 41.829,  *p* < 0.001* | χ^2^ = 24.586,  *p* < 0.001* |
| 60-69 | Males  N = 4,974 | χ^2^(2) = 101.177, *p* < 0.001*  Freq.: PCW = 1,308 (26%), ECW = 968 (19%), DCW = 1,141 (23%). | | |
|  |  | PCW vs. ECW | PCW vs. DCW | ECW vs. DCW |
|  |  | χ^2^ = 98.055,  *p* < 0.001* | χ^2^ = 23.372,  *p* < 0.001* | X^2^ = 27.469,  *p* < 0.001* |
|  | Females  N = 6,785 | χ^2^(2) = 366.935, *p* < 0.001*  Freq.: PCW = 2,747 (40%), ECW = 1,911 (28%), DCW = 2,270 (33%). | | |
|  |  | PCW vs. ECW | PCW vs. DCW | ECW vs. DCW |
|  |  | χ^2^ = 345.846,  *p* < 0.001* | χ^2^ = 113.915,  *p* < 0.001* | χ^2^ = 73.362,  *p* < 0.001* |
| 70-79 | Males  N = 4,422 | χ^2^(2) = 100.758, *p* < 0.001*  Freq.: PCW = 1,253 (28%), ECW = 926 (21%), DCW = 1,092 (25%). | | |
|  |  | PCW vs. ECW | PCW vs. DCW | ECW vs. DCW |
|  |  | χ^2^ = 95.830,  *p* < 0.001* | χ^2^ = 22.878,  *p* < 0.001* | χ^2^ = 28.478,  *p* < 0.001* |
|  | Females  N = 5,874 | χ^2^(2) = 196.202, *p* < 0.001*  Freq.: PCW = 2,602 (44%), ECW = 2,015 (34%), DCW = 2,281 (39%). | | |
|  |  | PCW vs. ECW | PCW vs. DCW | ECW vs. DCW |
|  |  | χ^2^ = 188.783,  *p* < 0.001* | χ^2^ = 56.233,  *p* < 0.001* | χ^2^ = 42.716,  *p* < 0.001* |
| 80+ | Males  N = 1,813 | χ^2^(2) = 31.281, *p* < 0.001*  Freq.: PCW = 576 (32%), ECW = 459 (25%), DCW = 543 (30%). | | |
|  |  | PCW vs. ECW | PCW vs. DCW | ECW vs. DCW |
|  |  | χ^2^ = 27.294,  *p* < 0.001* | χ^2^ = 2.129,  *p* = 0.145 | χ^2^ = 16.325,  *p* < 0.001* |
|  | Females  N = 2,877 | χ^2^(2) = 136.518, *p* < 0.001*  Freq.: PCW = 1,448 (50%), ECW = 1,097 (38%), DCW = 1,263 (44%). | | |
|  |  | PCW vs. ECW | PCW vs. DCW | ECW vs. DCW |
|  |  | χ^2^ = 128.812,  *p* < 0.001* | χ^2^ = 37.828,  *p* < 0.001* | χ^2^ = 31.510,  *p* < 0.001* |

* Significant

### Changes in trouble sleeping or recent change in sleep pattern across four waves (2yPCW, PCW, ECW and DCW)

| **Age group** | **Gender** | **Cochran’s Q test and McNemar test as Post Hoc analysis** | | |
| --- | --- | --- | --- | --- |
| 50-80+ | Males and females  N = 5,746 | χ^2^(3) = 254.687, *p* < 0.001*  Freq.: 2yPCW = 1,892 (33%), PCW = 1,959 (34%), ECW = 1,400 (24%), DCW = 1,645 (29%). | | |
|  |  | 2yPCW vs. PCW | 2yPCW vs. ECW | 2yPCW vs. DCW |
|  |  | χ^2^ = 2.914,  *p* = 0.088 | χ^2^ = 145.756,  *p* < 0.001* | χ^2^ = 36.699,  *p* < 0.001* |
|  |  | PCW vs. ECW | PCW vs. DCW | ECW vs. DCW |
|  |  | χ^2^ = 199.720,  *p* < 0.001* | χ^2^ = 64.623,  *p* < 0.001* | χ^2^ = 45.832,  *p* < 0.001* |

* Significant

### Changes in trouble sleeping or recent change in sleep pattern across four waves (4yPCW, PCW, ECW and DCW)

| **Age group** | **Gender** | **Cochran’s Q test and McNemar test as Post Hoc analysis** | | |
| --- | --- | --- | --- | --- |
| 50-80+ | Males and females  N = 21,356 | χ^2^(3) = 984.215, *p* < 0.001  Freq.: 4yPCW = 7,439 (35%), PCW = 7,777 (36%), ECW = 5,568 (26%), DCW = 6,601 (31%). | | |
|  |  | 4yPCW vs. PCW | 4yPCW vs. ECW | 4yPCW vs. DCW |
|  |  | χ^2^ = 19.023,  *p* < 0.001* | χ^2^ = 546.647,  *p* < 0.001* | χ^2^ = 111.025,  *p* < 0.001* |
|  |  | PCW vs. ECW | PCW vs. DCW | ECW vs. DCW |
|  |  | χ^2^ = 830.398,  *p* < 0.001* | χ^2^ = 237.221,  *p* < 0.001* | χ^2^ = 207.486,  *p* < 0.001* |

* Significant

## Changes in social parameters across three waves (PCW, ECW and DCW) or across two waves (ECW and DCW):

### Changes in current employment situation across three waves (PCW, ECW and DCW)

| **Age group** | **Gender** | **Cochran’s Q test and McNemar test as Post Hoc analysis** | | |
| --- | --- | --- | --- | --- |
| 50-80+ | Males and females  N = 31,268 | χ^2^(2) = 416.969, *p* < 0.001*  Freq.: PCW = 5,773 (18%), ECW = 4,970 (16%), DCW = 4,864 (16%). | | |
|  |  | PCW vs. ECW | PCW vs. DCW | ECW vs. DCW |
|  |  | χ^2^ = 254.734,  *p* < 0.001* | χ^2^ = 419.575,  *p* < 0.001* | χ^2^ = 4.208,  *p* = 0.040 |
| 50-59 | Males  N = 1,553 | χ^2^(2) = 175.728, *p* < 0.001*  Freq.: PCW = 1,145 (74%), ECW = 920 (59%), DCW = 1,071 (69%). | | |
|  |  | PCW vs. ECW | PCW vs. DCW | ECW vs. DCW |
|  |  | χ^2^ = 150.679,  *p* < 0.001* | χ^2^ = 26.645,  *p* < 0.001* | χ^2^ = 61.644,  *p* < 0.001* |
|  | Females  N = 2,769 | χ^2^(2) = 358.854, *p* < 0.001*  Freq.: PCW = 1,792 (65%), ECW = 1,392 (50%), DCW = 1,685 (61%). | | |
|  |  | PCW vs. ECW | PCW vs. DCW | ECW vs. DCW |
|  |  | χ^2^ = 298.129,  *p* < 0.001* | χ^2^ = 33.145,  *p* < 0.001* | χ^2^ = 151.986,  *p* < 0.001* |
| 60-69 | Males  N = 5,020 | χ^2^(2) = 185.349, *p* < 0.001*  Freq.: PCW = 1,289 (26%), ECW = 1,133 (23%), DCW = 947 (19%). | | |
|  |  | PCW vs. ECW | PCW vs. DCW | ECW vs. DCW |
|  |  | χ^2^ = 37.894,  *p* < 0.001* | χ^2^ = 198.432,  *p* < 0.001* | χ^2^ = 50.479,  *p* < 0.001* |
|  | Females  N = 6,798 | χ^2^(2) = 163.758, *p* < 0.001*  Freq.: PCW = 1,285 (19%), ECW = 1,163 (17%), DCW = 957 (14%). | | |
|  |  | PCW vs. ECW | PCW vs. DCW | ECW vs. DCW |
|  |  | χ^2^ = 21.219,  *p* < 0.001* | χ^2^ = 175.293,  *p* < 0.001* | χ^2^ = 58.859,  *p* < 0.001* |
| 70-79 | Males  N = 4,487 | χ^2^(2) = 54.749, *p* < 0.001*  Freq.: PCW = 135 (3.0%), ECW = 204 (4.5%), DCW = 116 (2.6%). | | |
|  |  | PCW vs. ECW | PCW vs. DCW | ECW vs. DCW |
|  |  | χ^2^ = 24.466,  *p* < 0.001* | χ^2^ = 2.769,  *p* = 0.096 | χ^2^ = 46.152,  *p* < 0.001* |
|  | Females  N = 5,889 | χ^2^(2) = 27.223, *p* < 0.001*  Freq.: PCW = 109 (1.9%), ECW = 130 (2.2%), DCW = 77 (1.3%). | | |
|  |  | PCW vs. ECW | PCW vs. DCW | ECW vs. DCW |
|  |  | χ^2^ = 3.604,  *p* = 0.058 | χ^2^ = 10.446,  *p* = 0.001* | χ^2^ = 24.360,  *p* < 0.001* |
| 80+ | Males  N = 1,865 | χ^2^(2) = 12.741, *p* = 0.002*  Freq.: PCW = 9 (0.5%), ECW = 21 (1.1%), DCW = 7 (0.4%). | | |
|  |  | PCW vs. ECW | PCW vs. DCW | ECW vs. DCW |
|  |  | Binomial distribution  *p* = 0.017 | Binomial distribution  *p* = 0.774 | Binomial distribution  *p* = 0.003* |
|  | Females  N = 2,887 | χ^2^(2) = 2.714, *p* = 0.257  Freq.: PCW = 9 (0.3%), ECW = 7 (0.2%), DCW = 4 (0.1%). | | |
|  |  | PCW vs. ECW | PCW vs. DCW | ECW vs. DCW |
|  |  | Binomial distribution  *p* = 0.774 | Binomial distribution  *p* = 0.180 | Binomial distribution  *p* = 0.453 |

* Significant

### Changes in feeling lonely across three waves (PCW, ECW and DCW)

| **Age group** | **Gender** | **Friedman test and Wilcoxon as Post Hoc analysis** | | |
| --- | --- | --- | --- | --- |
| 50-80+ | Males and females  N = 30,943 | χ^2^(2) = 229.394, *p* < 0.001*  Mean Rank: PCW = 1.97, ECW = 1.99, DCW = 2.04. | | |
|  |  | PCW vs. ECW | PCW vs. DCW | ECW vs. DCW |
|  |  | Z = -4.691,  *p* < 0.001* | Z = -14.604,  *p* < 0.001* | Z = -10.483,  *p* < 0.001* |
| 50-59 | Males  N = 1,547 | χ^2^(2) = 7.052, *p* = 0.029  Mean Rank: PCW = 1.99, ECW = 1.98, DCW = 2.03. | | |
|  |  | PCW vs. ECW | PCW vs. DCW | ECW vs. DCW |
|  |  | Z = -0.167,  *p* = 0.867 | Z = -2.185,  *p* = 0.029 | Z = -2.442  *p* = 0.015* |
|  | Females  N = 2,759 | χ^2^(2) = 18.486, *p* < 0.001*  Mean Rank: PCW = 1.98, ECW = 1.99, DCW = 2.04. | | |
|  |  | PCW vs. ECW | PCW vs. DCW | ECW vs. DCW |
|  |  | Z = -1.157,  *p* = 0.247 | Z = -3.882,  *p* < 0.001* | Z = -2.915,  *p* = 0.004* |
| 60-69 | Males  N = 4,960 | χ^2^(2) = 17.872, *p* < 0.001*  Mean Rank: PCW = 2.00, ECW = 1.98, DCW = 2.02. | | |
|  |  | PCW vs. ECW | PCW vs. DCW | ECW vs. DCW |
|  |  | Z = -1.875,  *p* = 0.061 | Z = -2.035,  *p* = 0.042 | Z = -4.016,  *p* < 0.001* |
|  | Females  N = 6,779 | χ^2^(2) = 55.966, *p* < 0.001*  Mean Rank: PCW = 1.96, ECW = 2.01, DCW = 2.03. | | |
|  |  | PCW vs. ECW | PCW vs. DCW | ECW vs. DCW |
|  |  | Z = -4.779,  *p* < 0.001* | Z = -7.185,  *p* < 0.001* | Z = -2.645,  *p* = 0.008* |
| 70-79 | Males  N = 4,397 | χ^2^(2) = 13.778, *p* = 0.001*  Mean Rank: PCW = 1.99, ECW = 1.99, DCW = 2.02. | | |
|  |  | PCW vs. ECW | PCW vs. DCW | ECW vs. DCW |
|  |  | Z = -0.279,  *p* = 0.780 | Z = -3.250,  *p* = 0.001* | Z = -3.551,  *p* < 0.001* |
|  | Females  N = 5,870 | χ^2^(2) = 103.620, *p* < 0.001*  Mean Rank: PCW = 1.95, ECW = 2.00, DCW = 2.06. | | |
|  |  | PCW vs. ECW | PCW vs. DCW | ECW vs. DCW |
|  |  | Z = -4.849,  *p* < 0.001* | Z = -10.249,  *p* < 0.001* | Z = -5.767,  *p* < 0.001* |
| 80+ | Males  N = 1,795 | χ^2^(2) = 18.390, *p* < 0.001*  Mean Rank: PCW = 1.97, ECW = 1.98, DCW = 2.05. | | |
|  |  | PCW vs. ECW | PCW vs. DCW | ECW vs. DCW |
|  |  | Z = -0.147,  *p* = 0.883 | Z = -3.412,  *p* = 0.001* | Z = -3.420,  *p* = 0.001* |
|  | Females  N = 2,837 | χ^2^(2) = 36.042, *p* < 0.001*  Mean Rank: PCW = 1.96, ECW = 1.98, DCW = 2.06. | | |
|  |  | PCW vs. ECW | PCW vs. DCW | ECW vs. DCW |
|  |  | Z = -1.257,  *p* = 0.209 | Z = -5.661,  *p* < 0.001* | Z = -4.637,  *p* < 0.001* |

* Significant

### Changes in face-to-face and electronic communication (combined) across three waves (PCW, ECW and DCW)

| **Age group** | **Gender** | **Friedman test and Wilcoxon as Post Hoc analysis** | | |
| --- | --- | --- | --- | --- |
| 50-80+ | Males and females  N = 29,595 | χ^2^(2) = 15,698.935, *p* < 0.001*  Mean Rank: PCW = 2.54, ECW = 1.72, DCW = 1.74. | | |
|  |  | PCW vs. ECW | PCW vs. DCW | ECW vs. DCW |
|  |  | Z = -101.360,  *p* < 0.001* | Z = -101.270,  *p* < 0.001* | Z = -3.055,  *p* = 0.002* |
| 50-59 | Males  N = 1,456 | χ^2^(2) = 1,229.124, *p* < 0.001*  Mean Rank: PCW = 2.69, ECW = 1.65, DCW = 1.66. | | |
|  |  | PCW vs. ECW | PCW vs. DCW | ECW vs. DCW |
|  |  | Z = -26.819,  *p* < 0.001* | Z = -26.750,  *p* < 0.001* | Z = -0.543,  *p* = 0.587 |
|  | Females  N = 2,646 | χ^2^(2) = 1,772.370, *p* < 0.001*  Mean Rank: PCW = 2.61, ECW = 1.68, DCW = 1.71. | | |
|  |  | PCW vs. ECW | PCW vs. DCW | ECW vs. DCW |
|  |  | Z = -33.008,  *p* < 0.001* | Z = -32.558,  *p* < 0.001* | Z = -1.654,  *p* = 0.098 |
| 60-69 | Males  N = 4,754 | χ^2^(2) = 3,737.698, *p* < 0.001*  Mean Rank: PCW = 2.67, ECW = 1.65, DCW = 1.68. | | |
|  |  | PCW vs. ECW | PCW vs. DCW | ECW vs. DCW |
|  |  | Z = -47.326,  *p* < 0.001* | Z = -47.591,  *p* < 0.001* | Z = -1.517,  *p* = 0.129 |
|  | Females  N = 6,512 | χ^2^(2) = 3,093.004, *p* < 0.001*  Mean Rank: PCW = 2.51, ECW = 1.73, DCW = 1.76. | | |
|  |  | PCW vs. ECW | PCW vs. DCW | ECW vs. DCW |
|  |  | Z = -44.789,  *p* < 0.001* | Z = -44.219,  *p* < 0.001* | Z = -1.895,  *p* = 0.058 |
| 70-79 | Males  N = 4,196 | χ^2^(2) = 3,083.821, *p* < 0.001*  Mean Rank: PCW = 2.65, ECW = 1.66, DCW = 1.69. | | |
|  |  | PCW vs. ECW | PCW vs. DCW | ECW vs. DCW |
|  |  | Z = -44.063,  *p* < 0.001* | Z = -43.824,  *p* < 0.001* | Z = -1.772,  *p* = 0.076 |
|  | Females  N = 5,604 | χ^2^(2) = 1,677.138, *p* < 0.001*  Mean Rank: PCW = 2.40, ECW = 1.80, DCW = 1.80. | | |
|  |  | PCW vs. ECW | PCW vs. DCW | ECW vs. DCW |
|  |  | Z = -34.491,  *p* < 0.001* | Z = -34.957,  *p* < 0.001* | Z = -0.334,  *p* = 0.739 |
| 80+ | Males  N = 1,719 | χ^2^(2) = 1,144.073, *p* < 0.001*  Mean Rank: PCW = 2.61, ECW = 1.69, DCW = 1.69. | | |
|  |  | PCW vs. ECW | PCW vs. DCW | ECW vs. DCW |
|  |  | Z = -26.790,  *p* < 0.001* | Z = -27.367,  *p* < 0.001* | Z = -0.420,  *p* = 0.675 |
|  | Females  N = 2,708 | χ^2^(2) = 578.406, *p* < 0.001*  Mean Rank: PCW = 2.34, ECW = 1.82, DCW = 1.84. | | |
|  |  | PCW vs. ECW | PCW vs. DCW | ECW vs. DCW |
|  |  | Z = -22.219,  *p* < 0.001* | Z = -22.108,  *p* < 0.001* | Z = -1.139,  *p* = 0.255 |

* Significant

### Changes in face-to-face communication across two waves (ECW and DCW)

| **Age group** | **Gender** | **Wilcoxon Signed-Rank test** |
| --- | --- | --- |
| 50-80+ | Males and females  N = 30,770 | Z = -81.856, *p* < 0.001*  Mean rank: ECW = 10,230.81, DCW = 13,737.51 |
| 50-59 | Males  N = 1,520 | Z = -14.880, *p* < 0.001*  Mean rank: ECW = 544.53, DCW = 679.19 |
|  | Females  N = 2,713 | Z = -25.131, *p* < 0.001*  Mean rank: ECW = 915.79, DCW = 1,223.49 |
| 60-69 | Males  N = 4,929 | Z = -32.143, *p* < 0.001*  Mean rank: ECW = 1,670.26, DCW = 2,224.46 |
|  | Females  N = 6,674 | Z = -43.160, *p* < 0.001*  Mean rank: ECW = 2,126.73, DCW = 3,030.77 |
| 70-79 | Males  N = 4,395 | Z = -30.727, *p* < 0.001*  Mean rank: ECW = 1,431.87, DCW = 1,947.29 |
|  | Females  N = 5,803 | Z = -35.921, *p* < 0.001*  Mean rank: ECW = 1,908.28, DCW = 2,563.69 |
| 80+ | Males  N = 1,844 | Z = -17.196, *p* < 0.001*  Mean rank: ECW = 623.48, DCW = 814.90 |
|  | Females  N = 2,892 | Z = -21.393, *p* < 0.001*  Mean rank: ECW = 999.66, DCW = 1,249.83 |

* Significant

### Changes in electronic communication across two waves (ECW and DCW)

| **Age group** | **Gender** | **Wilcoxon Signed-Rank test** |
| --- | --- | --- |
| 50-80+ | Males and females  N = 30,690 | Z = -9.594, *p* < 0.001*  Mean rank: ECW = 12,478.04, DCW = 11,970.86 |
| 50-59 | Males  N = 1,511 | Z = -2.281, *p* = 0.023*  Mean rank: ECW = 603.08, DCW = 607.25 |
|  | Females  N = 2,715 | Z = -2.325, *p* = 0.020*  Mean rank: ECW = 1,134.11, DCW = 1,070.12 |
| 60-69 | Males  N = 4,924 | Z = -3.024, *p* = 0.002*  Mean rank: ECW = 1,995.74, DCW = 1,930.75 |
|  | Females  N = 6,671 | Z = -1.679, *p* = 0.093  Mean rank: ECW = 2,667.93, DCW = 2,622.26 |
| 70-79 | Males  N = 4,376 | Z = -4.651, *p* < 0.001*  Mean rank: ECW = 1,799.91, DCW = 1,694.78 |
|  | Females  N = 5,802 | Z = -5.498, *p* < 0.001*  Mean rank: ECW = 2,365.26, DCW = 2,266.73 |
| 80+ | Males  N = 1,830 | Z = -4.588, *p* < 0.001*  Mean rank: ECW = 735.05, DCW = 696.64 |
|  | Females  N = 2,861 | Z = -3.666, *p* < 0.001*  Mean rank: ECW = 1,178.33, DCW = 1,086.28 |

* Significant
